# The Incorporation of Labile Protons into Multidimensional NMR Analyses: Glycan Structures Revisited

**DOI:** 10.1021/jacs.1c04512

**Published:** 2021-06-04

**Authors:** Mihajlo Novakovic, Marcos D. Battistel, Hugo F. Azurmendi, Maria-Grazia Concilio, Darón I. Freedberg, Lucio Frydman

**Affiliations:** † Department of Chemical and Biological Physics, Weizmann Institute of Science, 76100 Rehovot, Israel; ‡ Laboratory of Bacterial Polysaccharides, Center for Biologics Evaluation and Research, Food and Drug Administration, 10903 New Hampshire Avenue, Silver Spring, Maryland 20993, United States

## Abstract

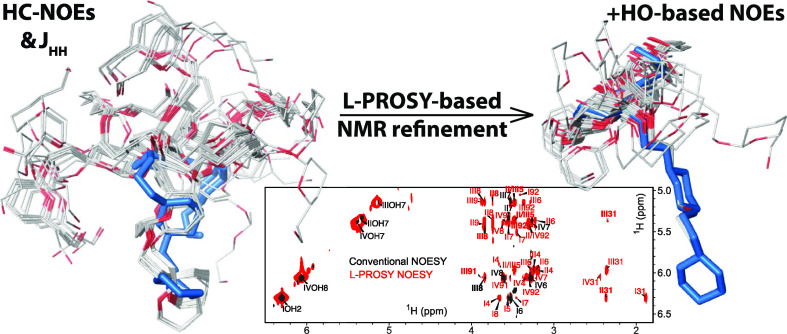

Glycan structures are often stabilized by a repertoire of hydrogen-bonded donor/acceptor groups, revealing longer-lived structures that could represent biologically relevant conformations. NMR provides unique data on these hydrogen-bonded networks from multidimensional experiments detecting cross-peaks resulting from through-bond (TOCSY) or through-space (NOESY) interactions. However, fast OH/H_2_O exchange, and the spectral proximity among these NMR resonances, hamper the use of glycans’ labile protons in such analyses; consequently, studies are often restricted to aprotic solvents or supercooled aqueous solutions. These nonphysiological conditions may lead to unrepresentative structures or to probing a small subset of accessible conformations that may miss “active” glycan conformations. Looped, projected spectroscopy (L-PROSY) has been recently shown to substantially enhance protein NOESY and TOCSY cross-peaks, for ^1^Hs that undergo fast exchange with water. This study shows that even larger enhancements can be obtained for rapidly exchanging OHs in saccharides, leading to the retrieval of previously undetectable 2D TOCSY/NOESY cross-peaks with nonlabile protons. After demonstrating ≥300% signal enhancements on model monosaccharides, these experiments were applied at 1 GHz to elucidate the structural network adopted by a sialic acid homotetramer, used as a model for α,2–8 linked polysaccharides. High-field L-PROSY NMR enabled these studies at higher temperatures and provided insight previously unavailable from lower-field NMR investigations on supercooled samples, involving mostly nonlabile nuclei. Using L-PROSY’s NOEs and other restraints, a revised structural model for the homotetramer was obtained combining rigid motifs and flexible segments, that is well represented by conformations derived from 40 μs molecular dynamics simulations.

## Introduction

Polysaccharides and glycans are ubiquitous in nature, acting both as integral constituents in numerous biomacromolecules and as additives in manufactured pharmaceuticals.^1−4^ Polysaccharides and glycans also pose a challenge when it comes to rationalizing their function in terms of structures or atomic-level dynamics: They tend to form amorphous solids unsuitable for X-ray diffraction, and their homopolymeric constitution and flexibility lead to a signal degeneracy that defies resolution by 1D NMR. High-dimensional NMR experiments can help lift this degeneracy and, therefore, are an integral part of their structure–function studies.^5−10^ Among the most common tools in these characterizations are nuclear Overhauser enhancement spectroscopy (NOESY), transferring polarization via dipolar interactions and shedding light on spatial proximities between ^1^Hs,^11−13^ and *J*-based magnetization transfers within a coupled network leading, via total correlation spectroscopy (TOCSY),^14−16^ to connectivity diagrams.^17,18^ Although hydroxyl protons (OH) are very valuable and contribute up to 50% of the observable glycan NMR signals, they undergo rapid chemical exchange with water, leading to reduced efficiency in their internuclear polarization transfers and thereby robbing these valuable reporters of a major role in 2D NMR studies. Thus, the long-range, nonsequential NOEs that are used to define a glycan’s 3D structure are usually limited to the aliphatic ^1^Hs of the polysaccharides. OH detection in glycans has been shown to help in hydrogen-bond (H-bond) detection, but these hydroxyl NMR signals could only be detected in nonphysiological solvents or under supercooled conditions, where exchange was slow.^10,19−22^

Recently, we have used projective measurements concepts^23,24^ to introduce new NOESY and TOCSY variants that successfully reinstated cross-peaks of rapidly exchanging sites in 2D NOESY/TOCSY NMR of proteins.^25^ The ensuing looped projective spectroscopy (L-PROSY) experiment alleviated the aforementioned problems by including the effects of chemical exchange. Indeed, instead of applying a single mixing period for isotropic transfers or cross-relaxation, L-PROSY “froze” these spins’ evolution while they were still proceeding with their rapid initial rates and used the exchange with the aqueous solvent to “reset” the initial conditions. By looping these projective *t*_1_-evolution measurements multiple times, this enabled cross-peaks to grow toward their maximum, thermodynamic limits. L-PROSY requires a suitable process that will reset the ^1^Hs sharing their magnetizations to their initial, maximum state. At room temperature, OH groups in saccharides exchange at ≈10–1000 s^–1^ with water, making them ideally suited to benefit from this procedure. In addition, it is desirable that the protons that are receiving the information from these labile sources remain undisturbed during the *t*_1_ encoding. L-PROSY measurements thus benefit from higher magnetic fields, which for small/medium-sized molecules improve resolution, while enabling the accommodation of a larger range of exchange rates and still retaining the identity of the labile site.

In view of these prospects, the present study extended these principles to examine the hydroxyl protons in saccharides and glycans. Enhancements about twice as large as previously observed in proteins were observed for these OHs; such enhancements led to the appearance of OH-derived cross-peaks at temperatures where conventional acquisitions fail to give discernible peaks. After fine-tuning the procedure on prototypical saccharides (myo-inositol, glucose, sucrose), NOESY and TOCSY L-PROSY experiments were focused on a sialic acid tetramer (Sia_4_). Sialic acid oligomers like Sia_4_ occur naturally and are linked through α,2–8 and/or α,2–9 glycosidic bonds; they are found on bacterial surfaces, on some tumors,^26,27^ in glycoproteins,^28^ attached to peptides in mucopolysaccharides, and are generally abundant, playing several physiological and pathological processes in mammals.^29^ α,2–8 polysialic acid (polySia) also comprises the bacterial capsules of *Neisseria meningitidis’* serogroup B, of *Escherichia coli* K1; it is also part of neural cell-adhesion molecules, as well as of many cancer cells.^30^ Although α,2–8 polySia is only weakly immunogenic, the reasons for this are poorly understood, as its closely related isomer α,2–9 polySia from *N. meningitidis* serogroup C elicits a strong immune response. Hydrogen-bonding patterns on Sia homopolymers were previously studied at subzero temperatures^5,31^ and using doubly labeled samples;^32^ based on the latter, it has been hypothesized that α,2–8 polySia’s 3D structure may help explain its distinct immunogenic properties. In this study, L-PROSY experiments carried out at 1 GHz allowed us to revise and improve previously derived structural motifs for Sia_4_, by providing additional information from correlations that exploit hydroxyl protons. These new correlations revealed longer-range, nonsequential NOE restraints at 5 °C and were augmented by further HSQC NOESY measurements at −10 °C, leading jointly to a set of conformations considerably more compact than hitherto assumed. The ensuing restraints also explained the heterogeneities observed in solvent exchange rates, in terms of the different hydrogen-bonded motifs. When incorporated into a simulated annealing algorithm, these restraints resulted in small root-mean-square deviations (RMSDs), and in the formation of new intersaccharide hydrogen-bonded structures, that could play a role in the biological recognition of sialosides.

## Results

### Targeting Labile ^1^Hs in Model Sugars: 2D and 3D NMR

**Figure 1:**
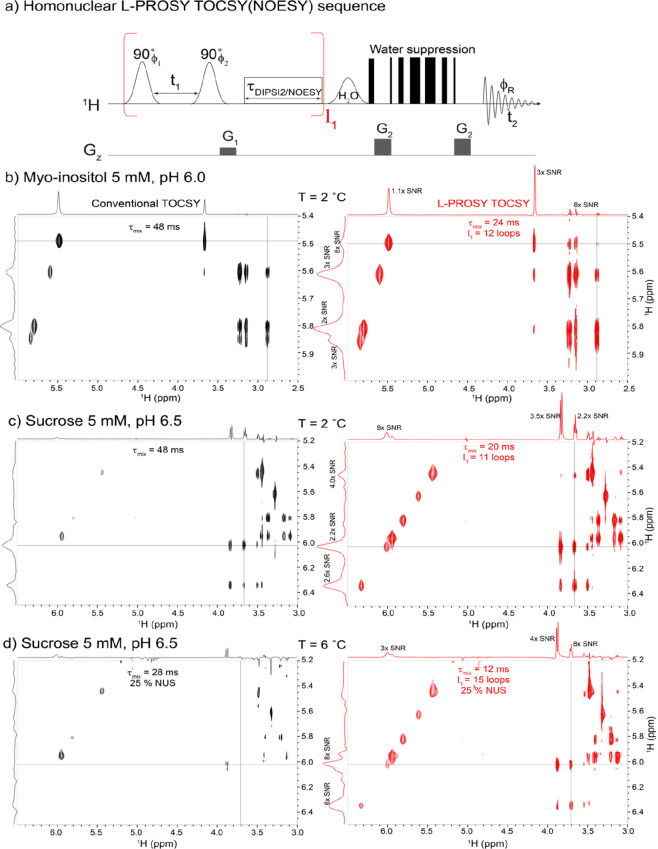
(a) Schematic L-PROSY TOCSY/NOESY sequence, with open shapes corresponding to frequency-selective 90° pulses addressing solely exchangeable sites (here the hydroxyl ^1^Hs), defining a *t*_1_ Ramsey modulation^33^ that is repeated together with a suitable mixing (delay for NOESY, DIPSI2^16^ for TOCSY) l_1_ times. This is followed by a water suppression scheme (flip-back followed by WATERGATE 3919^34,35^ or excitation sculpting^36^) and by the detection of all the resonances as a function of *t*_2_. A two- or four-step phase cycle was used: ϕ_1_ = 2(*x*, – *x*), ϕ_2_ = 2(*x*), 2(− *x*), and ϕ_R_ = *x*,2(− *x*), *x*. (b) L-PROSY TOCSY vs conventional TOCSY for a 5 mM myo-inositol sample at pH 6 and 2 °C. (c) Same as (b) but for a 5 mM sucrose sample at pH 6.5 and temperature of 2 °C. (d) Same as (c) but with the sample at 6 °C and using a 25% exponentially weighted NUS sampling^37−40^ to illustrate the compatibility of this procedure with L-PROSY. Shown on top are slices extracted at the indicated F_1_ positions (thin line). Notice how the faster exchange deteriorates the peaks in conventional experiments, while L-PROSY still provides high-quality spectra. With faster exchange rates, enhancements are ≥4× in L-PROSY, as predicted by the simulations in Supporting Figure S1. All spectra were acquired on a 600 MHz Bruker Avance III equipped with a Prodigy probe. The “streaks” visible in some of the aliphatic cross-peaks (particularly in the NUS acquisition) reflect the low contours chosen to depict the weaker OH resonances.

The L-PROSY principle as applied to homonuclear 2D TOCSY or NOESY is shown in Figure 1a. The experiment requires that labile protons be selectively addressed by a looped block, which includes both the *t*_1_ evolution and the mixing portion of the 2D NMR sequence. For the case of amide groups in proteins, this was facilitated by their relatively well-resolved nature. For polysaccharides, the choice of shaped pulses is more critical, as it needs to achieve a clean excitation (in both frequency and phase) and storage of the hydroxyls, that will leave unaffected the equilibrium polarization in the nearby water resonance. In this work, selective addressing was achieved using Q5 shaped pulses for both excitation and storage.^33^Figure 1b compares the resulting conventional and L-PROSY TOCSY spectra of myo-inositol at 600 MHz; equal resolutions for both spectra but an ≈5× average enhancement for the diagonal and cross-peaks in the L-PROSY data are observed. Figure 1c displays similar enhancements for a sucrose sample at 2 °C. At this temperature, the conventional TOCSY scheme still provides sufficient sensitivity for detecting several OH-aliphatic correlations; upon heating the sample by only 4 °C, however, the spectral quality deteriorates dramatically (Figure 1d). This reflects the averaging of the OH-aliphatic protons’ *J*-couplings effected by the faster chemical exchange between the hydroxyl and water protons, which prevents cross-peaks from developing. By contrast, L-PROSY still provides nearly all the cross-peaks that it revealed at lower temperatures, with enhancements vis-à-vis the conventional experiment about twice as large as those observed at 2 °C. This is a general behavior, where there is an interplay between the averaging that the chemical exchange imposes on the driving force (*J*-coupling or cross-relaxation) mediating the dialogue between the labile and nonlabile protons, and the chance of looping more often and in a more complete fashion, a labile polarization that is rapidly replenished by the chemical exchange. As a result of this, L-PROSY is endowed with only a weak dependence of the exchange rate, which, on the other hand, exponentially hurts the efficiency of conventional 2D correlations. A more detailed description of this phenomenon based on Liouville space calculations is presented in the Supporting Information (Supporting Figure S1 and associated discussion), which follows the L-PROSY enhancements for TOCSY experiments involving labile spins exchanging with the solvent and a nonlabile population. Also elaborated in Supporting Figure S1 is the fact that the relative L-PROSY enhancement will be larger for correlations driven by smaller *J*-coupling values. This enhancement heterogeneity is evidenced in the 5.5 ppm F_1_ slice shown in Figure 1b, where longer-range correlations defined by smaller *J*-values are enhanced ≥8× by L-PROSY acquisitions, while three-bond OH–CH cross-peaks are enhanced only ∼3-fold.

**Figure 2:**
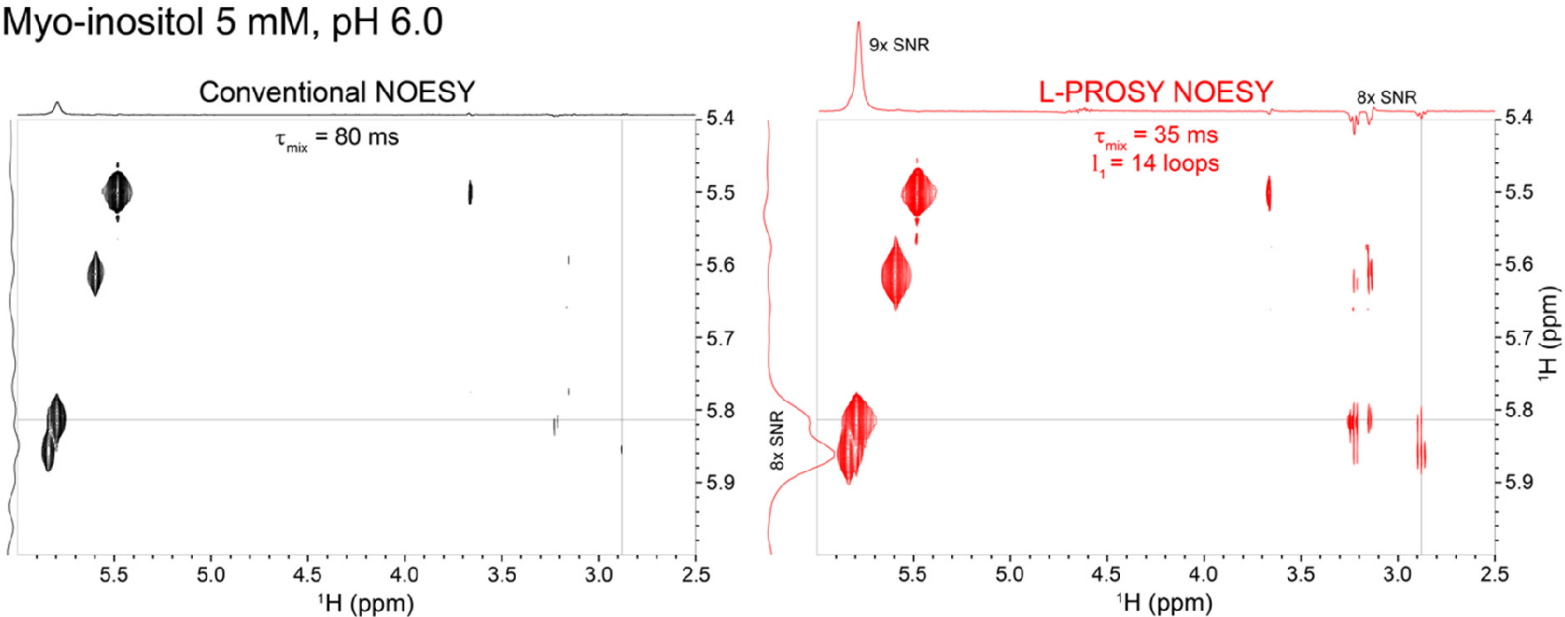
Comparison between conventional and L-PROSY NOESY experiments acquired using the indicated, individually optimized mixing parameters, on a 5 mM myo-inositol aqueous solution at 2 °C. Noted are the enhancements for the diagonal and cross-peaks at the indicated F_1_ slice; notice the negative cross-peaks arising from this molecule’s fast tumbling. Spectra were acquired on 600 MHz Bruker Avance III equipped with a Prodigy probe.

A theoretical analysis based on a Bloch–McConnell model^34−36^ shows a similar behavior arising when examining NOESY-based L-PROSY enhancements: Supporting Figure S2 and its associated paragraph show that up to an order of magnitude signal gains are achievable by L-PROSY NOESY, for realistic chemical exchange, cross-relaxation, and longitudinal relaxation rates. Figure 2 illustrates this with data, comparing conventional and L-PROSY NOESY correlations for myo-inositol. Cross-relaxation rates in small, fast tumbling molecules like myo-inositol are generally much smaller than usual *J*-coupling values; this opens up opportunities for large L-PROSY enhancements, comparable to those observed for long-range *J*-coupled partners. Indeed, in this specific case, L-PROSY provides ∼8-fold cross-peak NOESY enhancements as well as ∼9-fold gains for the diagonal peaks. Once again, L-PROSY capitalizes on rapid exchange with water to repolarize the labile protons, and relies on short, looped (14 × 35 ms long) mixing processes to achieve its gains.

**Figure 3:**
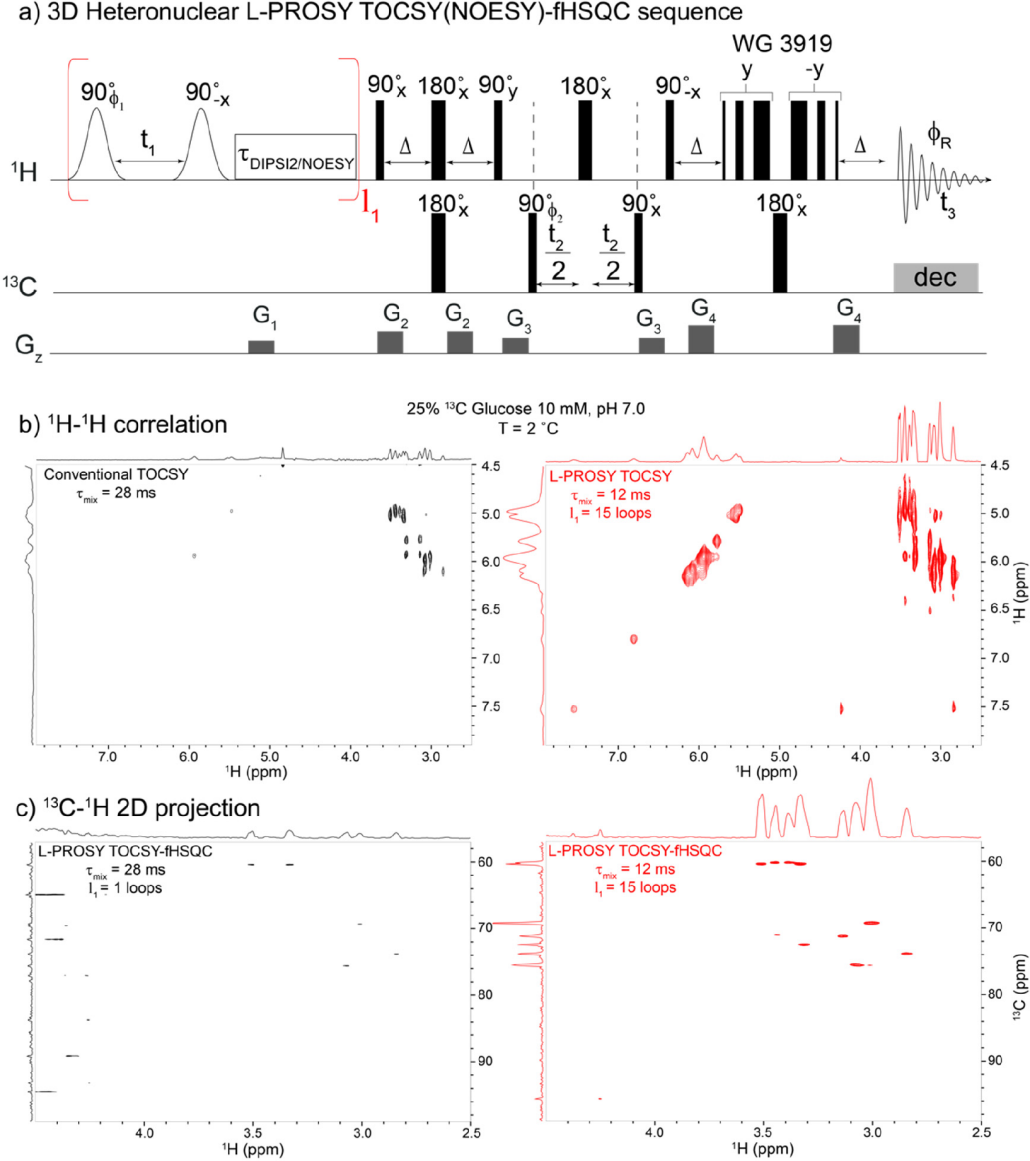
(a) 3D L-PROSY TOCSY (NOESY) fHSQC pulse scheme. Open shapes correspond to frequency-selective 90° excitation and storage pulses flanking the *t*_1_ evolution. Together with DIPSI2 (or NOESY) mixing periods, these are repeated l_1_ times and followed by an fHSQC scheme^37^ where ^13^C coherences evolve over *t*_2_. A two- or four-step phase cycle is used: ϕ_1_ = 2(*x*, – *x*), ϕ_2_ = 2(*x*), 2(− *x*), and ϕ_R_ = *x*,2(− *x*), *x*; offsets and bandwidths of the selective excitation/storage pulses are designed to address solely the hydroxyl ^1^H region and minimize perturbations on water and aliphatic ^1^H. The coherence transfer delay is Δ = 1/(4 × *J*_HC_). (b) Homonuclear L-PROSY TOCSY vs conventional TOCSY comparison recorded on a 10 mM, 25% ^13^C labeled glucose sample at pH 7 and *T* = 2 °C; mixing times were optimized for each acquisition scheme. (c) ^13^C–^1^H_C_ projection acquired using the sequence in (a) for conventional (black) and L-PROSY (red) correlations. Notice that all ^13^C sites of glucose are observed in the looped experiment, while many of them are missing in the conventional one. Observed enhancements are ≥4× in both homonuclear L-PROSY TOCSY and in the ^13^C-edited experiment. Shown on top of the 2D spectra are corresponding skyline projections.

The generality of these principles enables their incorporation into higher-dimensional experiments. Figure 3 exemplifies this, melding L-PROSY TOCSY with a fast HSQC block,^37^ in order to incorporate an additional ^13^C dimension, further resolving the aliphatic groups. The ensuing 3D experiment preserves both ^13^C-bound and aqueous protons longitudinally throughout most of its course, in order to minimize recovery delays. Homonuclear ^1^H–^1^H and heteronuclear ^1^H–^13^C projections acquired with conventional and L-PROSY versions of this experiment are illustrated in Figure 3b,c, respectively, for a 10 mM 25% randomly ^13^C-labeled glucose in a 90/10 H_2_O/D_2_O sample. These 2D projections reveal the preservation of L-PROSY’s ∼4–8-fold enhancements procedure, yielding a sensitivity that enables unambiguous H_O_-H_C_ assignments in this molecule. The full 3D correlation acquired using this L-PROSY TOCSY-fHSQC experiment is shown in Supporting Figure S3. Similar enhancements arise when these experiments are extended to NOESY-based correlations.

### Experimental Investigations of a Tetrameric Sialic Acid

**Figure 4:**
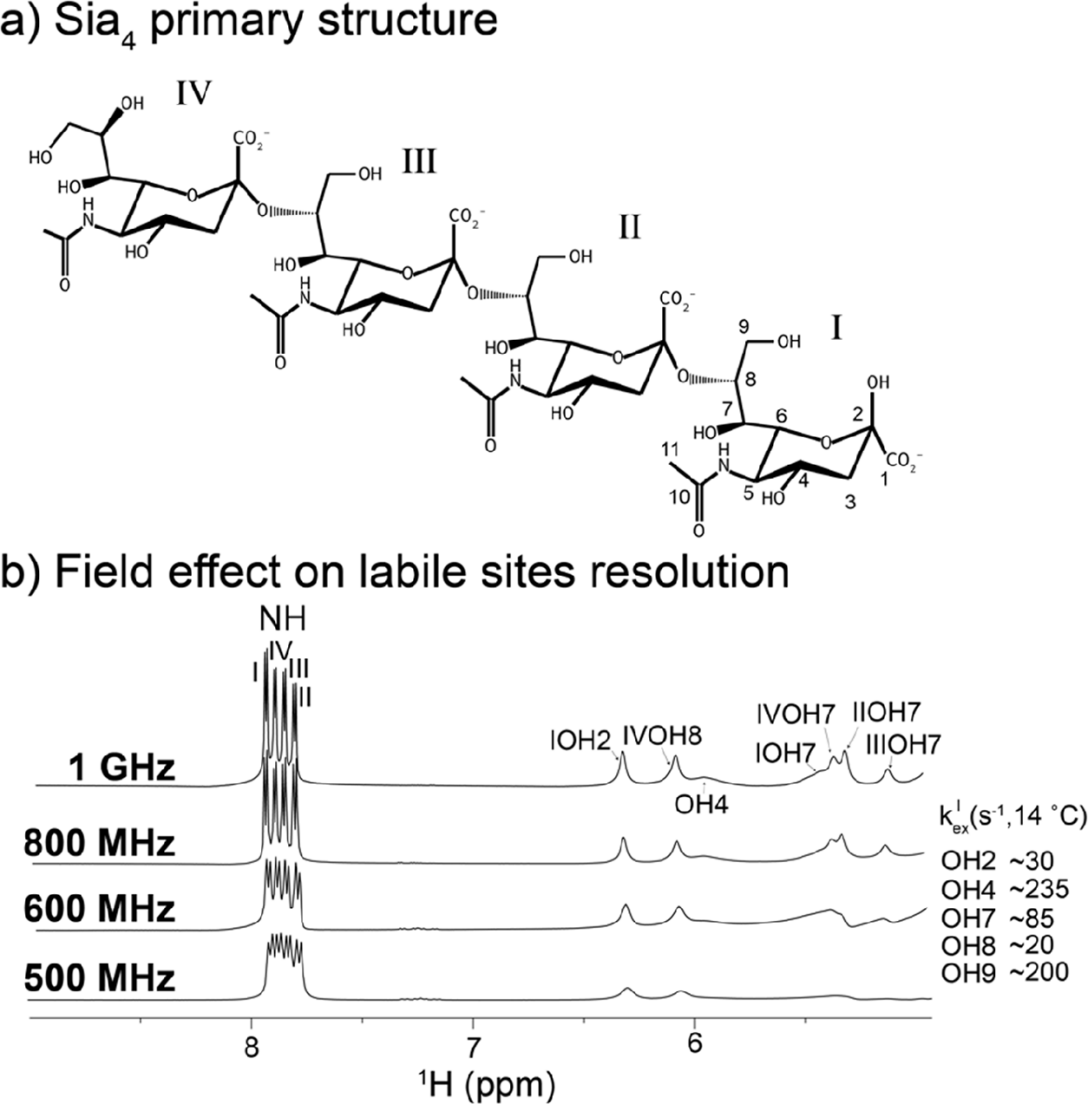
(a) Primary structure of the N-acetylated sialic acid tetramer (Sia_4_), linked through α,2–8 glycosidic bonds, used in this study, showing the atom numbering and ring nomenclature followed. (b) WATERGATE 3919 detection of Sia_4_’s labile hydroxyl and amide protons of at 14 °C at various magnetic fields; ≥1 GHz is necessary to resolve all amide sites together with their respective homonuclear couplings as well as most of the hydroxyl protons. Chemical exchange rates of these hydroxyl protons derived from results at −10 °C after accounting for thermal activation are shown in the inset. Fast exchanging OH4 and OH9 are broad and hard to detect even at high fields for this temperature.

With these tools in hand, we centered our attention on the sialic acid tetramer Sia_4_ (Figure 4a), a model system for the longer homopolymer α,2–8 polySia, whose elucidation could shed light on understanding how polySia adopts its 3D structure. The hydrogen-bonding network that can arise for N-acetylated polymers of this kind unlocks a diversity of possible stable, coexisting structures.^32^ However, OH-based hydrogen bonding in these glycans has not been explored directly, but inferred from monitoring NOEs involving C–H groups.^10,21,32^ In light of the results above, we applied L-PROSY to investigate the information arising from Sia_4_’s OH groups, aiming to study this oligomer at temperatures above the freezing point of pure water. To ameliorate the detrimental effects that proton exchange has on the signals of interest at these temperatures, we also resorted to using high-magnetic fields, which as shown in Supporting Figure S4, can significantly improve the spectral appearance of the hydroxyl protons. Thus, spectra recorded at 23.5 T (1 GHz ^1^H frequency; Figure 4b) and 14 °C resolve most OH ^1^H resonances, even if the OH4 peak at ≈6 ppm still remained substantially broad and OH9 remained unresolved and overlapped with OH7 protons due to its fast exchange rate (>200 s^–1^).^38^ Following these and other ancillary 2D tests, we decided to examine Sia_4_’s structure by collecting its NOE data at 1 GHz and 5 °C, conditions for which most of the OHs exhibit an intermediate exchange rate (Supporting Paragraph 4). Although not a physiological temperature, this is still 15 °C higher than what was required to obtain structural information in previous, lower field studies.^32^

**Figure 5:**
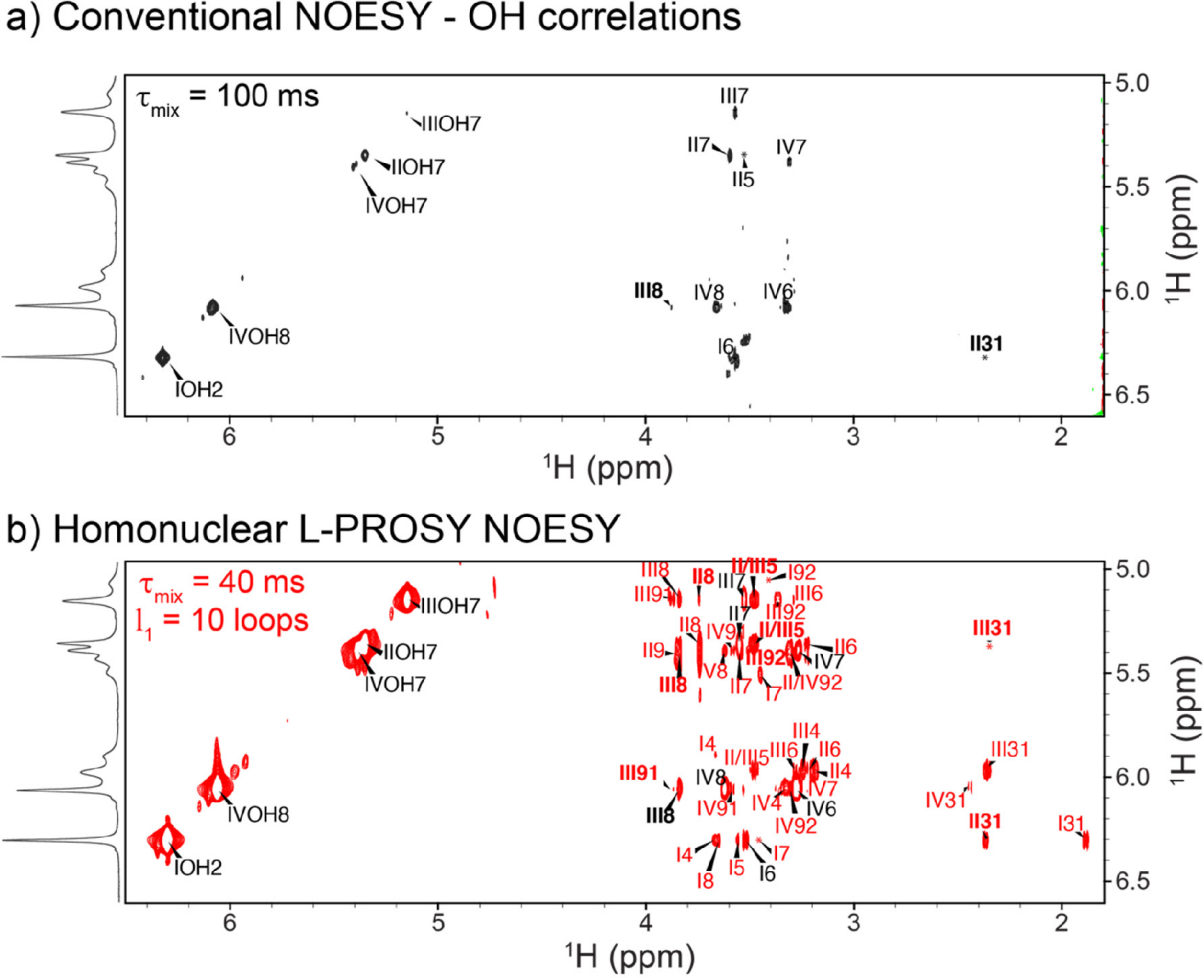
Conventional and L-PROSY NOESY experiments acquired on Sia_4_ at 5 °C and 1 GHz. Bold font indicates inter-residue cross peaks, and asterisks represent cross peaks that are present but not captured at the indicated contour levels. (a) Hydroxyl region of a conventional NOESY, optimized with a single 100 ms long mixing, which is the upper boundary when considering the fast OH’s chemical exchange with water; conventional NOESY shows only short-range OH-involved cross-peaks. (b) Homonuclear L-PROSY NOESY spectrum acquired under similar conditions, with 10 loops and 40 ms per loop, yielding an average enhancement of ∼4.5× vs the conventional NOESY as well as the multiple new long-range correlations labeled in red and presented in Table 1. Placed along the F_1_ axes are the hydroxyl proton regions acquired using 1D excitation sculpting at 5 °C and 1 GHz, as aids in the assignments. Roman and Arabic numerals reflect different rings and sites, per the convention in Figure 4a.

Figure 5 compares a conventional, optimized 1 GHz NOESY spectrum recorded on Sia_4_ at 5 °C, with its L-PROSY NOESY counterpart. Large enhancements are revealed in NOESY correlations involving the hydroxyl protons: Whereas the conventional spectrum shows few NOESY cross-peaks (Figure 5a), several longer-range NOEs are revealed by L-PROSY NOESY (red labels, Figure 5b). NOE enhancements are somewhat smaller (∼2.5-fold) when the same experiments are compared for the more slowly exchanging amide groups in this glycan (Supporting Figure S6). Still, for these protons, L-PROSY also enables inter-residue correlations that are otherwise absent in the conventional NOESY spectrum. A similar comparison for conventional TOCSY and L-PROSY TOCSY spectra acquired on Sia_4_ at 1 GHz is presented in Supporting Figure S5.

Glycan assignments can be greatly aided by ^13^C editing the NOESY and TOCSY cross-peaks.^38^ Thus, the L-PROSY strategy was integrated into HSQC schemes (Figure 3). A full assignment of the new inter- and intraresidue correlations arising from L-PROSY NOESY and NOESY-HSQC strategies for Sia_4_ at 5 °C is summarized in Table 1. To confirm that the new L-PROSY cross-peaks were solely of intramolecular origin, a series of diffusion experiments were performed in the relevant (5–160 mM) concentration range (see Materials and Methods and Supporting Figure S7), which revealed no aggregation. Conventional HSQC-NOESY experiments were also recorded on Sia_4_ at −10 °C; slowed OH chemical exchange rates allowed us to verify that the NOE correlations observed under these conditions were consistent with the L-PROSY-based NOESY collected at 5 °C (Table 1, Supporting Paragraph 8, and Figure S8). Table 1 highlights the increase in the number of OH-based NOEs observed at 5 °C when employing L-PROSY. The table also highlights the deleterious effect that OH exchange rates have on conventional NOESY, for the potential observation of long-distance correlations. For example, IIOH9, which exchanges rapidly with water, only provides a single short-distance intraresidue correlation in a conventional NOESY. By contrast, four correlations are detected for this hydroxyl proton with L-PROSY NOESY, including an inter-residue proton–proton long distance correlation.

**Table 1:**
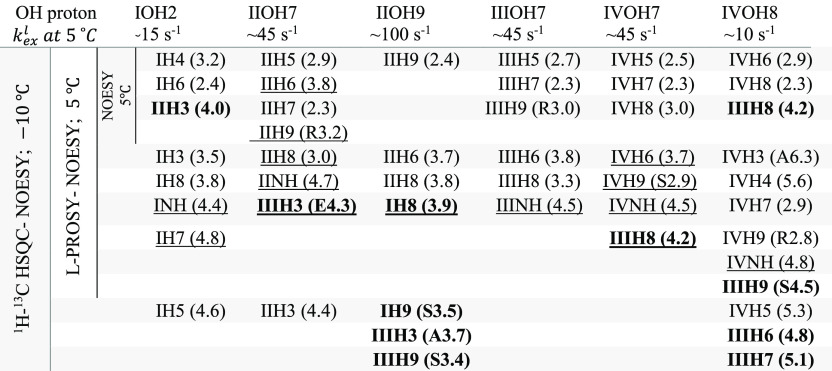
NOE Correlations Involving Hydroxyl Protons Detected in Conventional NOESY and L-PROSY NOESY Experiments at 5 °C, and in HSQC-NOESY correlations at −10 °C^a^

a Approximate chemical exchange rates for these hydroxyl protons are also shown. Inter-residue correlations are in bold, and underlined correlations are observed in NOESY and/or L-PROSY NOESY at 5 °C, but not in HSQC-NOESY at −10 °C. In parentheses are the ⟨*r*^–1/6^⟩^–1/6^ distances from MD at 5 °C. For H3A/E or H9R/S, the shortest distance is reported and indicated (both within deviation if not indicated).

To complement this NOE-based structural information, *J*-couplings for the glycerol chains connecting the residues were also measured using F2-^13^C-coupled HSQC, as previously reported^39^ (Table 2). The *J*-couplings that define ω_7_ (^3^*J*_H6,H7_) remain small (<2 Hz) and remarkably invariant for all linkages at −10 and 5 °C; this is consistent with (H6–C6–C7–H7) adopting torsion values of ≈ ± 90°. On the other hand, ^3^*J*_H7,H8_ (ω_8_) are similar for the glycerol chains of I and IV at both temperatures, and display larger values (ca. 7–9 Hz); this is consistent with H7,H8 being skewed toward an *anti* conformation. Conversely, ^3^*J*_H7,H8_ for residues II and III linkage displays values ca. 2–3 Hz; this may reflect a conformational average. Regarding the remaining ϕ/ψ torsion angle values that could be probed by NMR, only ψ (e.g., ^3^*J*_H8,C2′_) was readily accessible by NMR experiments on Sia_4_ samples in natural isotopic abundance. We also used PIP-HSQMBC^40^ to measure ^3^*J*_H8,C2′_, which yielded values of 2.9 ± 0.5, 4.2 ± 0.5, and 5.0 ± 0.5 Hz for residues I, II, and III, respectively (Supporting Figure S9). These values remained invariant within experimental error over the temperature range tested (−10, 5, and 37 °C), suggesting a constant conformational preference over that range.

**Table 2:**
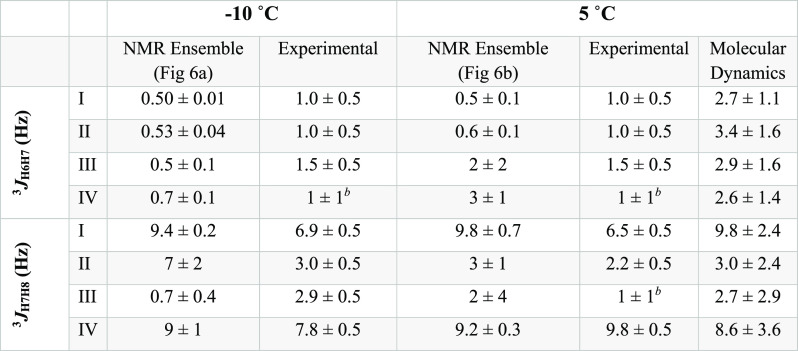
Experimental vs Theoretical ^3^*J*_HH_ Values Derived Either from NMR Structural Ensembles Torsion Angles (Figure 6) or the 40 μs MD Trajectory Using the Karplus-like Parameterization Described by Li et al. (ref (39))^a^

a ^3^*J*_HCCH_(ϕ) = 0.48 + 10.18 cos^2^(ω) – 0.03 cos(ω). The ^3^*J*_HH_ values derived from either the NMR ensembles or MD are reported as the mean value with their associated standard deviation. Errors for ^3^*J*_HH_ values measured by NMR reflect the spectral resolution used to collect the data.

b Peaks were broad and precluded precise coupling constant determinations.

### Sia_4_: Translating the NMR Insight into Structural Ensembles

With these −10 and 5 °C NMR data, simulated annealing (SA) together with the GLYCAM force field^41^ (see Materials and Methods) were used to derive structural ensembles for Sia_4_. The 5 °C structural derivations used the [O]H NOEs derived from L-PROSY as well as H[C] NOEs from HSQC-NOESY. To derive the structures at −10 °C, conventional NOEs, which included [O]H and H[C] NOEs, were used. In addition, six torsion angles derived from selected *J*-couplings were chosen to refine both ensembles, based on the fact that their values remained constant at both temperatures: ω_7_ for residues I, II, III, and IV and ω_8_ for residues I and IV. Torsions obtained from the restrained SA calculations (Supporting Figure S10) were converted to *J*-values^39^ for comparison to experimental data. Torsion angle values for ω_8_ (residues II and III) and ϕ/ψ for all residues were not restricted in the SA due to lack of evidence for defined torsions.

**Figure 6:**
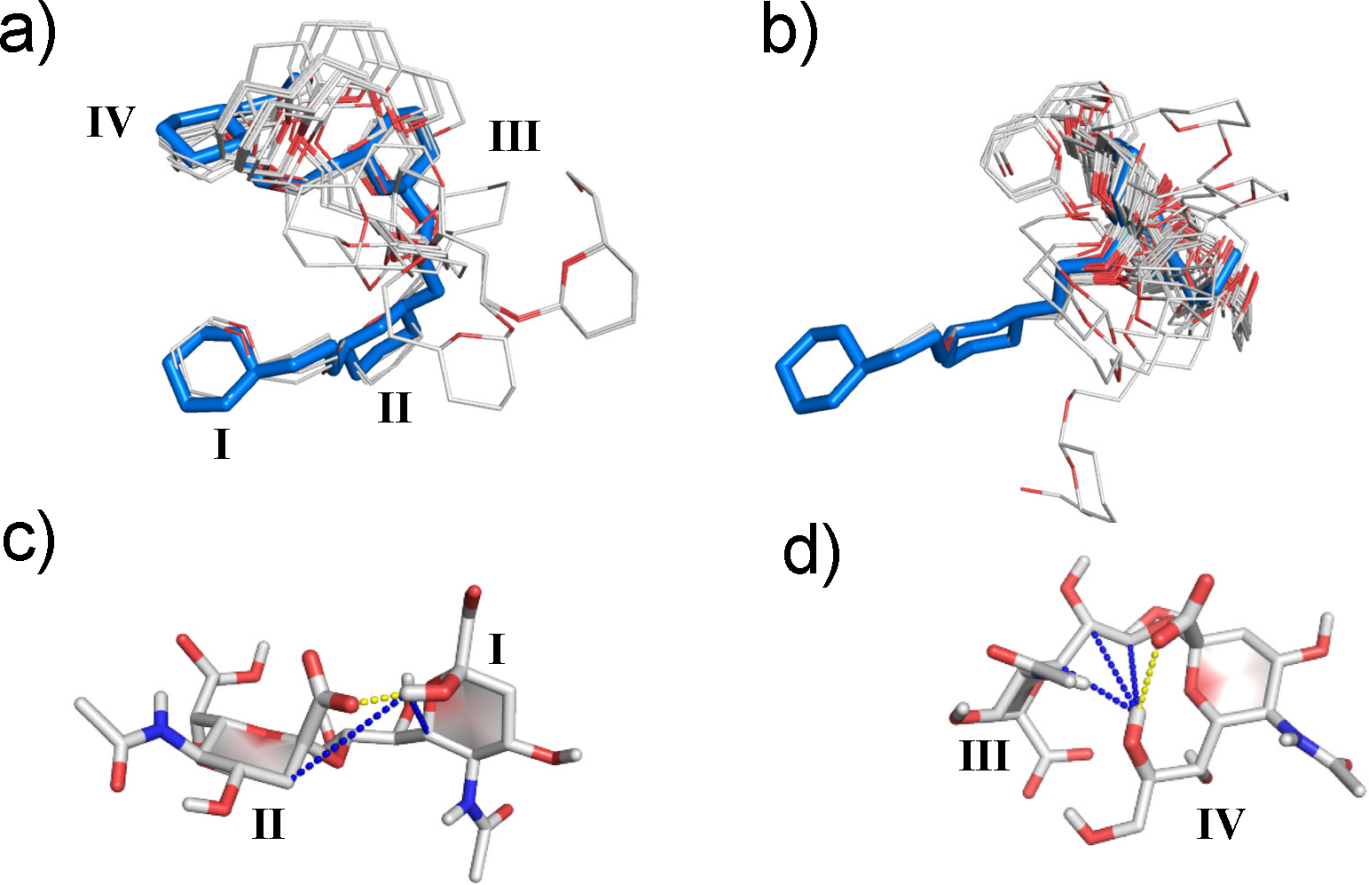
NMR ensembles composed of 100 structures generated via simulated annealing: Using restraints derived (a, c) from HSQC-NOESY at −10 °C and (b, c) from HSQC-NOESY/L-PROSY NOESY data at 5 °C (Table 1). Only the ring and linker chain heavy atoms are shown, with carbon and oxygen atoms depicted in white and red, respectively; close-to-mean structures are shown in blue. Residue numbering is indicated by Roman numerals, where “I” indicates the “reducing” and “IV” the “non-reducing” ends, respectively. Identified reducing and nonreducing end motifs present in Sia_4_ at −10 and 5 °C are presented in (c) and (d), respectively: Hydrogen bonds consistent with the NMR data are depicted by yellow dashed lines, and the key distances observed in both HSQC-NOESY and L-PROSY NOESY indicative of the resulting motifs are represented by blue dashed lines.

As shown in Figure 6, multiple possible conformations are consistent with the available NMR data at −10 and 5 °C. The computations show that Sia_4_ appears to adopt a compact structure (Figure 6a), approximately 9 ± 2 Å long (measured from IC2 to IVC6), with residues I and II adopting a well-defined conformation and the nonreducing end remaining undefined. The structural ensemble is in good agreement with experimental values, considering that structure-derived ^3^*J*_H7H8_ data for residues II and III in Table 2 represent ca. 68% confidence interval (restraints and statistics for the resulting structural models are detailed in Table S1). Despite the increase in the number of restraints for residues II and III, ω_8_ torsions do not show a preferred value, which could indicate the need of additional experimental restraints or an intrinsic dynamic behavior (see below).

In addition to these structural data, the NMR restraints suggest the presence of two persistent H-bonds: one between IOH2 and IIO1 (Figure 6c) and another between IVOH8 and IVO1 (Figure 6d). The presence of these H-bonds in Sia_4_ is supported by slow IVOH8 exchange rates,^38^ an unusual IVOH8 ^1^H chemical shift (deshielded ca. 0.5 ppm, compared to other non-anomeric ^1^HOs),^42^ small temperature coefficients for IOH2 and IVOH8 ^1^Hs chemical shifts (Supporting Figure S11), long-range IOH2 and IVOH8 NOEs, and the consistency of the data with that measured for Sia_2_, known to have a persistent IOH2/IIO1 H-bond.^43^ As for Sia_2_, the presence of the IOH2/IIO1 H-bond appears to contribute to the structural definition observed for the first two residues, while the IVOH8/IVO1 H-bond contributes to the conformation of residue IV’s glycerol chain. These portions of the molecule are characterized by small ^3^*J*_H6,H7_ and large ^3^*J*_H7,H8_ (Table 2), consistent with the well-defined ω_7_ and ω_8_ torsions in the resulting structural ensembles at −10 and 5 °C (Figure 6 and Supporting Figure S10).

**Figure 7:**
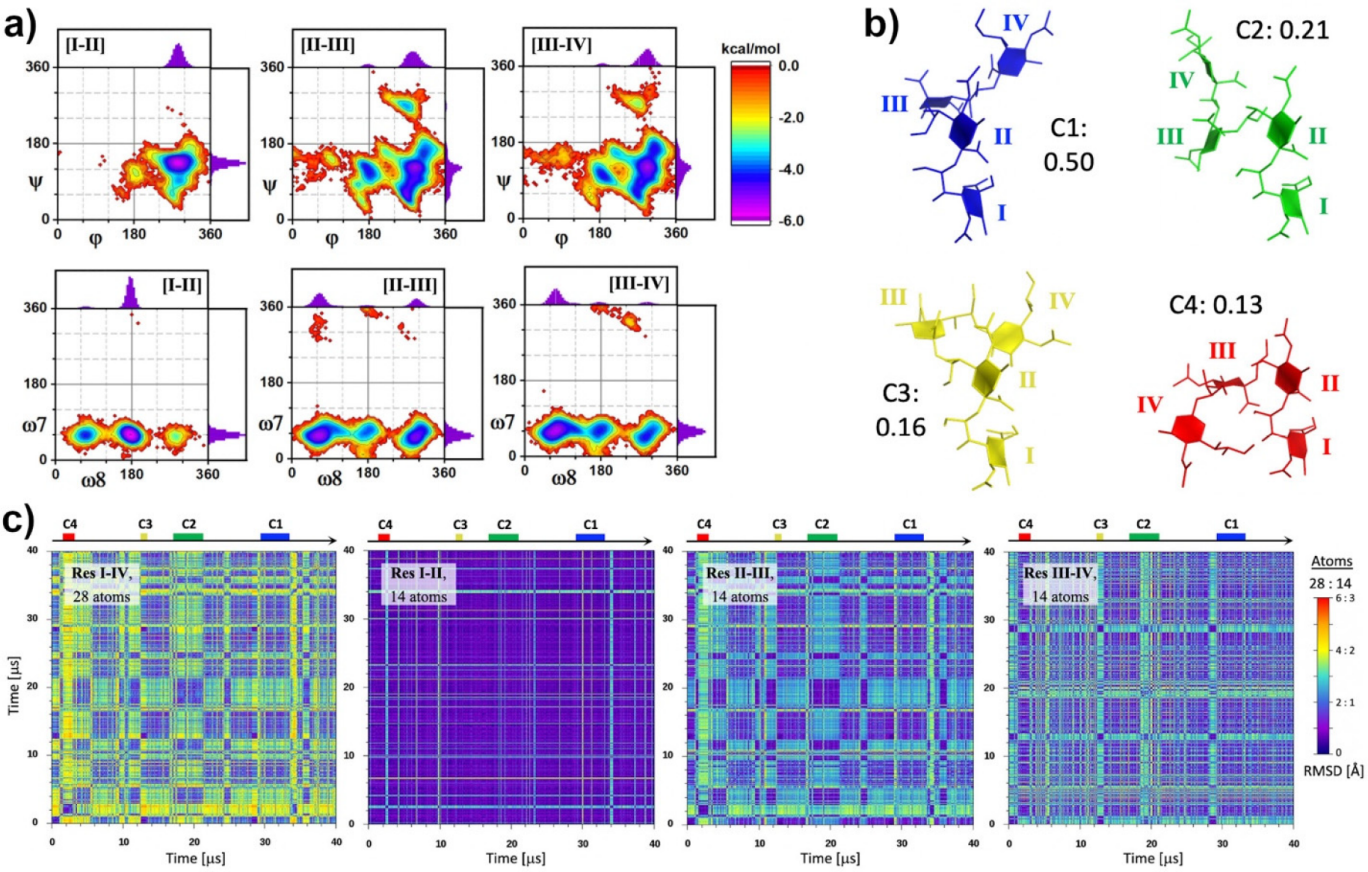
Summary of a 40 μs MD trajectory at 5 °C on Sia_4_. (a) Torsions (ϕ,ψ) and (ω_8_,ω_7_) energy maps for the three linkages in Sia_4_. The horizontal and vertical plots are population fractions for each torsion with bins of 5° size, the maximum fraction scale is 0.25. (b) Intermodel 2D RMSD maps for Sia_4_ with 28 atoms (C[1–6] + O6, residues I–IV) or 14 atoms (C[1–6] + O6, residues I–II, II–III, and III–IV, respectively), taking one frame every 10 ps. Each panel shows conformational similarities (RMSD < ∼2 or 1 Å, respectively) or dissimilarities (RMSD > ∼2 or 1 Å, respectively) in each case. (c) Representative conformations for the whole trajectory based on the 2D RMSD map with 28 atoms, any sampled Sia_4_ structure from the MD will result conformationally similar (RMSD < ∼2 Å) to only one of them in over 98% of the cases. The fraction of structures represented by each model is indicated; bars on top of the (b) panels point to representative clusters for each case. Models in (c) were RMSD[14 atoms]_res[1–2] to highlight the similarity of residues [I–II] conformations throughout.

Additional insight for the interpretation of the measured NMR observables, was sought from extended, unrestrained molecular dynamics (MD) simulations. Several conditions were tried (see Materials and Methods) before settling on a 40 μs computation at 5 °C. The conformations sampled by Sia_4_ throughout this MD simulation yielded average NMR values that were in good agreement with experimental HSQC-NOESY data (−10 °C) and L-PROSY (5 °C) OH NOEs (Table 1, values in parentheses) as well as with many of the measured ^3^*J*_HH_ at both temperatures (Table 2). As observed for the NMR-derived SA ensembles, MD predicts a well-defined and persistent conformation for residues I–II and higher flexibility for residues III–IV, as reflected by the well-defined energy wells for [I–II] links in the torsion energy maps, and much bigger spread with multiple minima for the [II–III] and [III–IV] links (Figure 7a). To identify potential representative structural motifs in this trajectory, pairwise 2D-RMSD analyses were performed for over 4000 models from the 40 μs trajectory. Attention was centered on the four saccharide residues (C[1–6] + O6 positions, 28 atoms) and on two-residue groupings (residues I–II, II–III, and III–IV; 14 atoms each); results of these analyses are shown in Figure 7b. The analysis for residues I–IV predicts an interconversion among multiple loosely persistent conformations, identified by alternating regions of low and high RMSD values when looking across rows or columns for a given time frame. Detailed inspection suggests four relatively independent clusters of conformationally stable regions, based on a 2 Å RMSD cutoff between structures. Although there are exceptions, these represent ∼2% of the 400,000 structures analyzed, scattered randomly over the trajectory. Representative time periods for each cluster are indicated by colored horizontal bars on top of each plot (one example for each cluster, for clarity). To extract representative models for each of these clusters, averaged structures from 11 contiguous frames within each region were taken, and RMSD values for all frames were compared against the same 28 atoms in each averaged reference set. Figure 7c shows models with low RMSD values for each case, where the model colors and labels correspond to the horizontal bars on Figure 7b. Also indicated is the fraction that each of these representations has, relative to the whole trajectory. Figure 7b also plots the 2D RMSDs for pairs of residues in the oligomer. These plots highlight the conformational stability of residues I–II on Sia_4_, for almost the whole trajectory, as low and continuous pairwise RMSDs are evidenced (short-lived departures notwithstanding). On the other hand, for both pairs of residues II–III and III–IV, alternating cycles of low and high RMSD values can be identified. There is also an apparent difference in the change rates of the II–III pair, whose changes appear slower than for the III–IV pair. Consequently, the 2D RMSD for the tetramer as a whole seems to be dominated by the dynamics of residues III and IV, relative to the two first residues.

## Discussion

This study demonstrates that L-PROSY can substantially enhance NOESY and TOCSY cross-peaks at minimal cost in experimental complexity when targeting hydroxyl groups in saccharides. These OH groups tend to undergo fast chemical exchanges with the solvent, leading to even larger losses than amides in proteins when targeted in 2D homonuclear correlations. At the same time, the L-PROSY enhancing effect becomes larger, thanks to a more rapid and complete repolarization of the source spins. These gains are not monotonic with exchange rates (Supporting Figures S1 and S2), as extensive chemical exchange with water eventually averages away the identity of the targeted OH sites as well as the cross-couplings linking labile and nonlabile sites. The former loss can be attenuated by increasing the spectral separation among the labile and other sites by increasing the external field, something that also facilitates L-PROSY’s use of selective pulses.

The experiments presented here demonstrate these effects on simple model compounds and on the more complex oligosaccharide Sia_4_, whose structure could be reexamined at 5 °C using newly obtained L-PROSY correlations. Although this temperature is not physiological, it is a meaningful 15 °C higher than the previous study utilizing labile ^1^Hs.^32^ The new data suggest that earlier attempts to reduce polySia models to neatly defined helices with different pitches^32,44−47^ may have been premature, as the derived models from NMR-based restrained SA and long duration MD point to complex dynamics together with an interplay of subtle interactions. For instance, NOEs from the labile ^1^Hs in the L-PROSY NOESY spectra at 5 °C challenge a proposed extended helical model (Supporting Figure S12), as the resulting distances for that model for IIOH7-IIIH3 (6.3 Å), IIOH9-IH8 (9.7 Å), IVOH7-IIIH8 (6.5 Å), IVOH8-IIIH8 (6.9 Å), and IVOH8-IIIH9 (6–7 Å) would fall beyond the experimental limits. The difference between the SA models in Figure 6a,b and the previously proposed extended helical model is not surprising either, as the latter was generated assuming three persistent intraresidue N5H···O8 H-bonds, whose existence may have been unwarranted.^32^ The resulting SA structures of Sia_4_ with inclusion of the novel long-range restraints led to the more compact ensembles shown in Figure 6a,b, which produce relatively long average distances for the N5H–O8 in residues I, II, and III (3.68 ± 0.04, 3.5 ± 0.8, and 4.1 ± 0.4 Å at −10 °C and 4.0 ± 0.1, 5.0 ± 0.2, and 4.8 ± 0.7 Å at 5 °C, respectively; see Supporting Figure S13), stressing the low probability of these atoms forming H-bonds. A similar conclusion was recently presented against the extended helix model based mainly on theoretical calculations,^48^ while L-PROSY allows access to the required experimental support for a similar outcome.

The NMR data collected at both −10 and 5 °C are consistent with a well-defined conformation adopted by the reducing end residues (I and II) and a less defined nonreducing end (III–IV). Gaining further insight required resorting to MD computations, extending much longer durations than previously attempted. Preferred conformations then began to emerge in simulations at 5 °C when runs extended to 40 μs. This allowed us to identify four independent conformational models based on RMSD of the ring atoms, as representative for the great majority of trajectory structures. MD also predicted molecular flexibility that explained the lack of resolved features in the NMR structural ensembles.

The models derived for Sia_4_ from both experimentally restrained SA and MD are characterized by two prominent structural motifs, one at each end of the molecule. At the reducing end, a H-bond between IOH2···IIO1 appears to dictate the conformation adopted by the first two residues (Figure 6c; Supporting Figures S10 and S13). This motif had already been observed for the shorter Sia_2_ homologue and was predicted to be a common feature of α,2–8-linked Sia oligomers;^43^ this could alter the dynamic properties of oligomers with the reducing end as β-sialic acid (preferred configuration in aqueous solution), compared to α-sialic acid. For the nonreducing end, we observed not only a high potential flexibility but also the presence of a stable motif proposed to exist for terminal sialic acids irrespective of the type of linkage (Figure 6d).^49^ This second motif is defined by the conformation adopted by the glycerol chain of the terminal end residue (IV), characterized by a IVOH8···IVO1 H-bond (yellow dash, Figure 6d). Importantly, a direct through-hydrogen-bond correlation was observed via long-range HSQMBC between IVOH8 and IVC1, consistent with a persistent IVOH8···IVO1 H-bond (Supporting Figure S9b). Interestingly, this IVOH8···IVO1 H-bond is consistent with the nonreducing end of the folded structure found in the X-ray structure of Sia_8_ bound to an antibody fragment of mAb735 expressed in *E. coli*, where the nonreducing end terminal motif characterized by an IVOH8···IVO1 H-bond is also present.^50^ Also worth noting, the reported H-bonds for Sia_4_ are supported by slow OH exchange rates, even at 5 °C (Table 1). Confirming the presence of this nonreducing end motif could have important implications, since it represents a key source of structural similarities and differences between α,2–8 (*N. meningitidis* serogroup B, Men B) vs α,2–9 (*N. meningitidis* serogroup C, Men C) polysaccharides. On one hand, the nonreducing end terminal residue for both Men B and Men C polysaccharides may lead to equivalent exposed epitopes. On the other hand, while Men B has only one free OH8 at the nonreducing end, Men C has one for every linker between residues that could lead to the formation of H-bonds that stabilize a different set of conformations. Moreover, the presence or lack of such H-bonds in Men C could shed light on the observed distinct immunogenic properties between *O*-acetyl (+) and *O*-acetyl (−)Men C, since O-acetylation at C8 would lead to conformational change by disrupting the hydrogen-bonded motif.

## Conclusions

This work demonstrates some of the advantages of introducing OH-related correlations to improve the chances of studying increasingly challenging glycans, enabling new 2D and 3D NMR variants to deliver information closer to physiological conditions and thus improving predictions of glycan containing vaccines and therapeutics. Several additional systems could benefit from the signal enhancements and the structural data that can be accessed in this manner. Foremost among these are nucleic acids that have, in addition to OHs, rapidly exchanging imino and amino protons. Enhancements could also arise in experiments involving nuclei affected by paramagnetic and/or quadrupolar interactions, which can replenish their polarization even if no solvent chemical exchange is present. The applicability of L-PROSY on these labile and nonlabile sites is being explored.

Using this and other tools, attention was focused on the Sia_4_ oligomer as a test model for a complex oligosaccharide. With the aid of high fields, well-defined structural elements could thus be established over a range of temperatures and used in simulated annealing structural calculations. The accessibility to new NMR observables at 5 °C enabled us to evaluate the reliability of MD predictions to support and help interpret our results. These led to well-defined, convergent motifs for the reducing and nonreducing ends of the glycan. These structural features uncovered for Sia_4_ may be “diluted”, as the oligosaccharide chain becomes longer. Still, the study of smaller polymer fragments like Sia_4_ helped to uncover structural features in this kind of polymer that may otherwise have been obscured by the signatures and behavior of middle residues. This warrants further investigations into the structure, dynamics, and function of these important systems, in longer, more complex polysaccharides.

## Materials and Methods

### Sample preparation

d-Glucose (U–^13^C_6_, 24–25%) was purchased from Cambridge Isotope Laboratories, and a 10 mM sample was prepared in H_2_O/D_2_O (90%:10%) at pH 7.0. The sucrose sample was prepared using household sugar at a concentration of 5 mM and pH 6.5, whereas the same concentration of myo-inositol was made at pH 6.0, both in H_2_O/D_2_O (90%:10%). Unlabeled α,2–8 Sia_4_ was purchased from Nacalai Inc. (San Diego, CA). 25 mg of α,2–8 sialic acid tetramer were dissolved in 250 μL (78.7 mM) of 20 mM phosphate buffer at pH 6.5, containing H_2_O:D_2_O (90%:10%) and 0.05% NaN_3_. The 33% ^13^C- and 100% ^15^N-labeled Sia_4_ sample was purified as previously described;^32^ however, for its bacterial growth, 33% of ^13^C-labeled glucose was used. A 32 mM solution was made similarly in 250 μL of 20 mM phosphate buffer at pH 6.5, containing H_2_O:D_2_O (90%:10%) and 0.05% NaN_3_. Both samples were placed in a Shigemi tubes. For data collected at −10 °C, a 78.7 mM Sia_4_ at natural isotopic abundance, containing 0.1% DSS and H_2_O:D_2_O (90%:10%), pH 7.5 was prepared and placed in a Wilmad tube for data collection. For hydrogen-bond detection, the Sia_4_ concentration was increased to 157.4 mM.

### NMR Spectroscopy

NMR experiments on myo-inositol, sucrose, and glucose were conducted on a 14.1 T (600 MHz) Avance III Bruker spectrometer equipped with a liquid-nitrogen-cooled “Prodigy” probe. The multidimensional Sia_4_ experiments, except where otherwise noted, were recorded at 23.5 T (1 GHz) using an Avance NEO console and three different probes. L-PROSY experiments in Supporting Figures S5b and S4 were recorded with a 5 mm room-temperature RT-TXI probe. L-PROSY NOESY experiments in Figure 5b as well as the standard NOESY and TOCSY experiments shown in Figure 5a and Supporting Figure S5a used a TCI CryoProbe. To test the magnetic field effects on hydroxyl protons resolution, additional 1D spectra were acquired at 11.7 T (500 MHz) using a Magnex magnet interfaced with a Varian iNova console (Palo Alto, USA) on a triple resonance HCN Varian 5 mm probe and on an 18.8 T (800 MHz) Avance III Bruker spectrometer equipped with a TCI CryoProbe. Conventional TOCSY experiments were acquired using dipsi2gpph19/dipsi2esgpph standard Bruker sequences using a DIPSI2 isotropic mixing,^16,51^ while for NOESY experiments, noesyfpgpph19/noesyesgpph^52−54^ was used. In all cases, optimal mixing times and optimal WATERGATE delays for binomial water suppression according to the magnetic field were used. Another important ingredient was the use of Q5 pulses^33^ in all L-PROSY NOESY/TOCSY experiments, as these provide the cleanest excitation and maximum preservation of water. Bandwidths and offsets of these pulses were optimized prior to every experiment using Ramsey excite-store modulation scheme with the goal to maximize OH/NH signal and minimize water (typically bandwidths 1.5–3 ppm that are 1.7–3.0 ms long depending on the NMR external magnetic field as well, with offsets at least 1 ppm away from water). All data collected at −10 °C were acquired on a 16.4 T (700 MHz) Avance III Bruker spectrometer equipped with a XYZ-gradient TCI CryoProbe.

### Vicinal Homonuclear Coupling Constants Determination

These were determined from coupled HSQC experiments (Bruker sequence hsqcetgpsi with minor modifications) employing the weakly coupled ^13^C satellite for (^3^*J*_HH_) between, H6,H7 and H7,H8 of each Sia residue at 37, 25, 5, and −10 °C.^55−58^

### Probing Sia_4_ Aggregation by Translational Diffusion

Diffusion ordered spectroscopy (DOSY) experiments (Figure S7) were performed to probe for α,2–8 Sia_4_ aggregation in the 5–160 mM concentration range. DOSY experiments were acquired utilizing the Bruker pulse sequence ledpgp2pr. Translational diffusion constants at 5 and −10 °C were obtained using TopSpin3.6’s diffusion analysis module, by fitting the peak area vs gradient strengths curves. Each translational diffusion constant was calculated using five different spectral regions of α,2–8 Sia_4_ and tabulated as a mean ± standard deviation. Because DSS only yields a single peak, only a single diffusion constant per temperature was determined and used to obtain the ratio of translational diffusion coefficient value ± fitting error. The translational diffusion ratio reflects the molecules’ hydrodynamic radii ratio (D_DSS_/D_Sia_4__= Rh_Sia_4__/Rh_DSS_).

### Data Processing

All NMR data were either processed using Bruker Topspin or NMRPipe software^59^ with the SMILE plugin.^60^ Usually both dimensions were apodized using QSINE/SINE or exponential window function, zero-filled once, deconvoluted from the residual water signal, and phased to an absorption mode. Spectra illustrating comparison between L-PROSY and conventional methods are plotted at the same noise level. Numerical simulations were carried out using a home-written Matlab code for Bloch–McConnell calculations or the Spinach package.^61,62^

### Simulated Annealing for NMR Ensemble Structure Determination

Simulations were performed using the Amber 16 software package in a workstation equipped with four GeForce GTX 780 3GB GPUs. The Sia_4_ structure and parameter files were created using the Glycam Biomolecule builder tool available online (www.glycam.org) and the Glycam-06h force-field.^41^ NMR distance restraints used for structure calculations were obtained from assigned 150 ms HSQC-NOESY experiments collected at −10 and 5 °C. Spectral assignment was carried out in CCPNMR Analysis.^63^ 100 independent simulated annealing (SA) runs were performed utilizing each set of NMR-derived restraints: one set of restraints collected at 5 °C and two sets of restraints from data collected at −10 °C. To derive the structure ensemble at 5 °C, NOE restraints for aliphatic protons (from HSQC-NOESY experiments) and labile ^1^H (derived from L-PROSY at 5 °C) as well as torsion angle restraints were used. To derive the structure ensemble at −10 °C, restraints were obtained from: (1) data from Battistel et al.^32^ plus torsion angles derived from newly parametrized Karplus-like relation;^39^ and (2) NOE restraints for aliphatic and labile ^1^H as well as torsion angle restraints. For NOE-derived restraints, the calculated distance ±1 Å was used. For torsion angle restraints, the target value ±30° was used. For the degenerate torsion angles defined by H6–C6–C7–H7 (±90°), both potential values were allowed. The SA protocol was utilized as follows: The temperature was maintained at 300 K for 3 ps, gradually increased, and maintained at 1500 K over 197 ps, and the system temperature was then decreased first to 300 K for 50 ps and subsequently cooled to 0 K for 50 ps. The NMR-derived restraints were turned on during the cooling period and increased gradually, reaching 100% (32 kcal/(mol × Å)) force at 250 ps into the run. To ensure no ring distortions occur due to the high temperatures used in the SA protocol, torsion angles for maintaining chair ring conformations were introduced as restraints. The resulting structures were screened for violations (distance, angles, torsions, and ring distortions). If violations were found, the conflicting restraints were isolated and subjected to further analysis. Once no violations were obtained, the resulting structures were ranked by energy and ordered from 1 to 101 in the NMR ensemble. All the structures in the resulting for each NMR bundle were aligned to the minimum energy structure using the C and O (ring and glycosidic) atoms of residues I to II. Structure grouping, alignment, and measurements were carried out in PyMOL (The PyMOL Molecular Graphics System, Version 2.1, Schrödinger, LLC.).

### Molecular Dynamics

MD simulations were performed using the AMBER 18 software package^64^ in a workstation equipped with four GeForce GTX 1080Ti Graphic Processing Units. The Sia_4_ structures and parameter files were created using the XLEaP module included in AMBER with the force field Glycam-06h.^41^ The initial conformer was charge-neutralized with 4 Na^+^ counterions and solvated with 1436 TIP3FB explicit water models.^65^ A cubic cell of approximate size 35 Å by side with periodic boundary conditions was used. Nonbonded van der Waals and electrostatic scaling factors for 1–4 interactions were set to unity (SCEE = SCNB = 1) as required by Glycam. Long-range electrostatic interactions were computed with particle-mesh Ewald summation,^66^ with a nonbonded cutoff distance of 8 Å. The initial system was energy minimized. Then a 1 ns preparation MD run was used to equilibrate the system to the target temperature (TT) for production, as follows: 0 to 323 K in 0.1 ns, 323 K from 0.1 to 0.6 ns, 323 K to TT from 0.6 to 0.8 ns, TT from 0.8 to 1.0 ns. The results discussed in this manuscript are for MD runs of 40 μs at TT = 278 K, with NPT conditions and hydrogen mass repartition^67^ to allow an integration time for the equations of motion of 4 fs, with hydrogen-containing covalent bonds constrained to their equilibrium lengths using the SHAKE algorithm.^68^ MD production choices were selected utilizing shorter test runs (between 1 and 10 μs) exploring the effect of various parameters like water model (TIP3P), TT, HMR, and cell size, to ensure that the conclusions derived from MD were not affected by these choices.

Processing of trajectories was performed using *cpptraj*, included in AMBER, and python in-house scripts. For analysis, H-bonds were geometrically defined with cutoff values for donor–acceptor distances (*d*_DA_) of 3.0 Å and angles (θ_D-H-A_) of 135°. The four representative structural models of the 40 μs trajectory were selected using a 2D RMSD plot of 4000 models (1 every 10 ps), using the ring atoms plus C1 positions (i.e., C[1–6] + O6) for all residues. Arbitrary time points were selected from four unique clusters, and average RMSD reference models (with 28 atoms) were created for each frame ±5, totaling 11 consecutive models. Then, RMSD values were calculated for 400,000 models of the 40 μs trajectory against each reference to obtain the fraction population representations given in Figure 7c. Figures were created with OriginPro 2019 (www.OriginLab.com; Northampton, MA), PyMol (www.schrodinger.com/pymol), Gnuplot (www.gnuplot.info) and GIMP (www.gimp.org).

## Supporting Information Available

Theoretical analyses of L-PROSY TOCSY and L-PROSY NOESY enhancements for hydroxyl groups; L-PROSY 3D TCSY-fHSQC correlations; magnetic field dependence of L-PROSY enhancement in glycans; L-PROSY vs conventional TOCSY correlations in Sia_4_; L-PROSY NOESY and NOESY-fHSQC NMR on Sia_4_’s amide ^1^Hs; diffusion NMR experiments to probe Sia_4_’s aggregation; HSQC-NOESY results on Sia_4_ at −10 °C; *J*_CH_ measurements on Sia_4_; simulated annealing results on Sia_4_; Sia_4_ OH’s chemical shift temperature dependence; HSQMBC-based H-bond detection experiments; and structural ensemble statistics derived at different temperatures for Sia_4_ (PDF)
