# Supplementary material for: The Incorporation of Labile Protons into Multidimensional NMR Analyses: Glycan Structures Revisited
Source: J Am Chem Soc. 2021 Jun 4;143(23):8935–48. doi: 10.1021/jacs.1c04512 (PMC8297728; doi:10.1021/jacs.1c04512)
Supplement: Supplementary file 1 — ja1c04512_si_001.pdf [file ja1c04512_si_001.pdf]

Supporting Information for

The incorporation of labile protons into multidimensional NMR analyses: Glycan structures, revisited

Mihajlo Novakovic[1], Marcos D. Battistel[2], Hugo F. Azurmendi[2], Maria-Grazia Concilio[1], Darón I. Freedberg[2] and Lucio Frydman[1,*]

[1]Department of Chemical and Biological Physics, Weizmann Institute of Science, 76100 Rehovot, Israel

[2]Laboratory of Bacterial Polysaccharides, Center for Biologics Evaluation and Research, Food and Drug Administration, 10903 New Hampshire Ave, Silver Spring, Maryland, United States 20993

*Email: lucio.frydman@weizmann.ac.il

***1) A numerical analysis of L-PROSY TOCSY enhancements in saccharides*.** Total correlation spectroscopy (TOCSY) experiments rely on magnetization transfers over a period of extended duration under a suitable average mixing Hamiltonian $\bar{\mathcal{H}}^m$. This mixing strives to eliminate all chemical shifts from $\bar{\mathcal{H}}^m$, leaving mutual spin-spin couplings establishing an "isotropic mixing" Hamiltonian:[1]

$$\bar{\mathcal{H}}^m = \sum_{i<j} 2\pi J \boldsymbol{I_i I_j} \quad (1)$$

Eigenfunctions of $\bar{\mathcal{H}}^m$ involve collective spin modes that are responsible for the coherence/magnetization transfers. Because the Hamiltonian is isotropic, all three components of the magnetization (*x,y,z*) behave identically. In the absence of chemical exchange, evolution of the x-component from spin 1 to spin 2 in a two-spin system can be explicitly shown as[1]

$$I_{1x} \to I_{1x}\frac{(1+\cos 2\pi J t_{mix})}{2} + I_{2x}\frac{(1-\cos 2\pi J t_{mix})}{2} + (I_{1y}I_{2z} - I_{1z}I_{2y})\sin 2\pi J t_{mix} \quad (2)$$

To describe TOCSY experiments in exchanging systems containing several spins that interact through scalar couplings, magnetization treatments based on classical Bloch-McConnell equations are not valid. Therefore, we resort to a full density-matrix treatment that includes relaxation and chemical exchange superoperators. The Liouvillian superoperator[2,3] used in these spin simulations thus contained Zeeman (chemical shift) terms for the labile proton (M_l) and the water (M_{H_2O}) as well as for the non-labile (M_{nl}), a scalar *J*-coupling between the latter two protons assumed to be on the saccharide, an ideal spin-lock responsible for establishing the effective isotropic Hamiltonian, relaxation within an extended T_1/T_2 approximation, and chemical exchanges between the labile proton and water. The ensuing master equation[1]

$$\frac{d}{dt}\boldsymbol{\rho} = \left(-i\widehat{\boldsymbol{\mathcal{H}}} - \widehat{\boldsymbol{\Gamma}} + \widehat{\boldsymbol{\Xi}}\right)\boldsymbol{\rho} + \widehat{\boldsymbol{\Gamma}}\boldsymbol{\rho}^{\mathbf{0}} \quad (3)$$

where $\widehat{\mathcal{H}}$, $\hat{\boldsymbol{\Gamma}}$ and $\hat{\boldsymbol{\Xi}}$ are Hamiltonian, relaxation and exchange superoperators respectively, was solved numerically using custom-written codes within the Spinach package.[4–6]

Supporting Figure S1 illustrates results from these simulations for TOCSY and L-PROSY TOCSY experiments. Incoherent chemical exchanges with the solvent average out homonuclear *J*-couplings, dampening the coherent oscillation in Eq. 2 and reducing the efficiency of TOCSY experiments. Supporting Figure S1 illustrates this: blue dashed lines represent the fast decay of a labile diagonal peak in a conventional TOCSY experiment, while red and green dashed lines show the buildups of corresponding cross-peaks for *J*-couplings equal to 7 Hz (typical for three-bond coupling values) and 2 Hz (typical for longer-range couplings), respectively. Using same color code, solid lines represent the outcome of the L-PROSY TOCSY experiment. Notice the substantially higher cross-peak intensities that the latter can achieve, yielding enhancements ε of ≈2-3x for larger *J*-couplings and slower exchange rates, and ≈3-5x for long-range couplings and/or fast exchange rates.

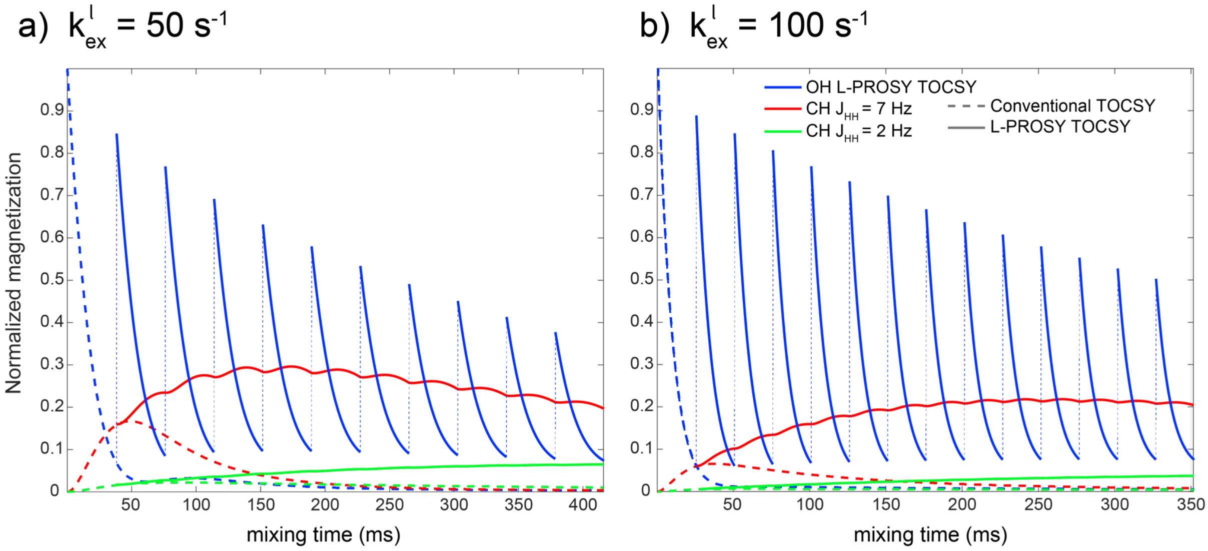

Supporting Figure S1. Trajectories of labile and non-labile protons' spin-locked magnetization during conventional TOCSY and L-PROSY TOCSY experiments when chemical exchange with solvent is a) 50 s^{-1} and b) 100 s^{-1}. Dashed lines represent conventional TOCSY experiments while solid lines describe the evolution during L-PROSY experiments. To simulate the conventional TOCSY experiment, a CW irradiation is applied after initial excitation of labile hydroxyl proton (using perfect delta function pulse). Dashed blue curves show the fast decay of the diagonal peak due to relaxation and chemical exchange, not allowing cross-peaks (red for 7 Hz and green for 2 Hz *J*-coupling) to build up. The L-PROSY experiment is simulated in a similar manner, except that short CW isotropic mixing periods were interleaved with multiple selective excitation pulses on the labile proton. This in turn utilizes fast chemical exchange repolarization to provide multiple transfers of magnetization to non-labile spin yielding much stronger cross-peaks. In all simulations the aqueous pool was assumed ~150-fold larger than the solute (enough to reliably simulate chemical exchange decoherent processes); both longitudinal and transverse relaxation times were chosen to be: $T_{1\rho}^{l} = 0.3\ s$, $T_{1\rho}^{nl} = 0.6\ s$ and $T_{1\rho}^{H_2O} = 0.7\ s$ exemplifying relaxation parameters in rotating frame of a small molecule.

In general, when choosing optimal L-PROSY TOCSY parameters, two generalizations can be made: the line widths of the labile protons report on the chemical exchange rates and

their inverse can be used to estimate the mixing time per loop, while the number of loops l_l is chosen based on maximum memory time of non-labile protons represented by relaxation in rotating frame: $l_1 \times t_{mix} \sim T_{1\rho}^{nl}$.

2) Numerical analysis of L-PROSY NOESY enhancements in saccharides. To examine the applicability of L-PROSY in fast exchanging hydroxyls like those targeted in this study, a classical multi-site Bloch-McConnell-Solomon equations model was utilized, focusing on the evolution of various longitudinal magnetization components throughout repeated NOE mixing periods. Three different spin pools were assumed in our model, comprising the magnetizations of labile protons (M_l), water (M_{H_2O}) and non-labile protons (M_{nl}):

$$\frac{d}{dt}\begin{pmatrix} M_{nl} \\ M_l \\ M_{H_2O} \end{pmatrix} = \begin{pmatrix} -\frac{1}{T_1^{nl}} - \sigma & \sigma & 0 \\ \sigma & -\frac{1}{T_1^l} - \sigma - k_{ex}^l & k_{ex}^{H_2O} \\ 0 & k_{ex}^l & -\frac{1}{T_1^{H_2O}} - k_{ex}^{H_2O} \end{pmatrix} \begin{pmatrix} M_{nl} \\ M_l \\ M_{H_2O} \end{pmatrix} + \begin{pmatrix} \frac{M_{nl}^0}{T_1^{nl}} \\ \frac{M_l^0}{T_1^l} \\ \frac{M_{H_2O}^0}{T_1^{H_2O}} \end{pmatrix} \quad (4)$$

T_1^l, T_1^{nl} and $T_1^{H_2O}$ in Eq. (4) are longitudinal relaxation times in the case of NOESY experiment and M_l^0, M_{nl}^0 and $M_{H_2O}^0$ are the equilibria magnetizations of the corresponding spin reservoirs. Labile protons are allowed to undergo chemical exchange with the water, while connected to a non-labile spin pool that is receiving polarization via a generic cross-relaxation[7] process represented by a rate σ. In order to include population differences between the aqueous and solute water pools exchange rates were also scaled according to:

$$k_{ex}^l[solute] = k_{ex}^{H_2O}[H_2O] \quad (5)$$

For the specific instance of a L-PROSY NOESY experiment, σ represents the difference between zero- and double-quantum dipole-dipole cross-relaxation rates, and can be expressed in terms of normalized spectral densities $\mathcal{J}$ as

$$\sigma = \frac{1}{10} b^2\big(\mathcal{J}(0) - 6\mathcal{J}(2\omega^0)\big) \quad (6)$$

where $\mathcal{J}(\omega) = \frac{\tau_c}{1+\omega^2\tau_c^2}$ and $b = -\frac{\mu_0}{4\pi}\frac{\hbar\gamma^2}{r^3}$ is the dipole-dipole coupling constant.[8]

To simulate the effect of L-PROSY's looping, numerical propagations of Eq. (4) were repeated a number of times, while setting up new initial magnetization conditions for each repetition equaling the final magnetizations arising from the previous propagation. Furthermore, two sets of simulations were carried out to simulate the *x* / *-x* phase-cycling

involved in L-PROSY experiments: one started from $M_0 = \begin{pmatrix} 1 \\ 1 \\ 1 \end{pmatrix}$; the other from $M_0 = \begin{pmatrix} 1 \\ -1 \\ 1 \end{pmatrix}$ and performed an inversion of the final M_l before repeating each loop in order to mimic the effects of the phase cycling. The magnetizations arising from each propagation were then subtracted, and normalized with respect to those stemming from a conventional (non-looped, optimized) NOESY. Supporting Figures S2a and S2b show the predicted enhancements, calculated as $\varepsilon = M_{nl}(t_{mix}, l_1)/M_{nl}(t_{mix}^{opt}, l_1 = 1)$, as a function of mixing time and of number of loops, upon assuming different exchange rates. Notice that according to these surface plots, the faster the exchange rate the higher the L-PROSY enhancements that can be achieved. This reflects the rapid decay in the efficiency of the conventional NOESY, rather than an actual absolute increase of the cross-peaks arising in L-PROSY. Supporting Figure S2c extends this analysis by showing how L-PROSY enhancements vary as function of the cross-relaxation rate dictated by rotation tumbling time τ_C between the labile and non-labile protons, for different chemical exchange rates. For all τ_C values enhancements increase monotonically with chemical exchange, reaching over an order of magnitude improvement for exchange rates of ~50 s^{-1}. The effects of the relaxation of the labile and non-labile protons is examined in Figure S2d, which shows that enhancements rise monotonically as the relaxation of the non-labile protons becomes slower. While this T_1^{nl} sets up an upper boundary for the ε, T_1^l barely influences the looped transfer: only for very short values of T_1^l enhancements are affected and become ~20% higher. Two effects contribute to this improvement: a less efficient transfer in the conventional experiment, and a faster effective relaxation of the labile protons that enables a faster, more complete L-PROSY looping (akin to having a faster k_{ex}). The effects of the non-labile proton relaxation parameters on the cross-peak buildup, leads to a simple correlation between the number of loops l_1 and the mixing time t_{mix} that can be useful when choosing optimal acquisition parameters:

$$\varepsilon(NOESY): l_1 \times t_{mix} \sim T_1^{nl}; \tag{7}$$

On the other hand, the optimal mixing time t_{mix} will be determined by the rate of chemical exchange, which should be fast enough to provide sufficient repolarization of the labile protons over its course: $t_{mix} \approx (k_{ex})^{-1}$. This clarifies why higher enhancements are achieved in faster exchanging systems: this exchange effectively allows for shorter mixing times until labile protons recover, and consequently more loops can be applied. Given that the NOESY experiment generally requires longer mixing times than TOCSY but that at the same time it

depends on (the longer) T_1 rather than on $T_{1\rho}$, more loops will be feasible and thus larger L-PROSY enhancements will usually be achieved for this kind of correlations.

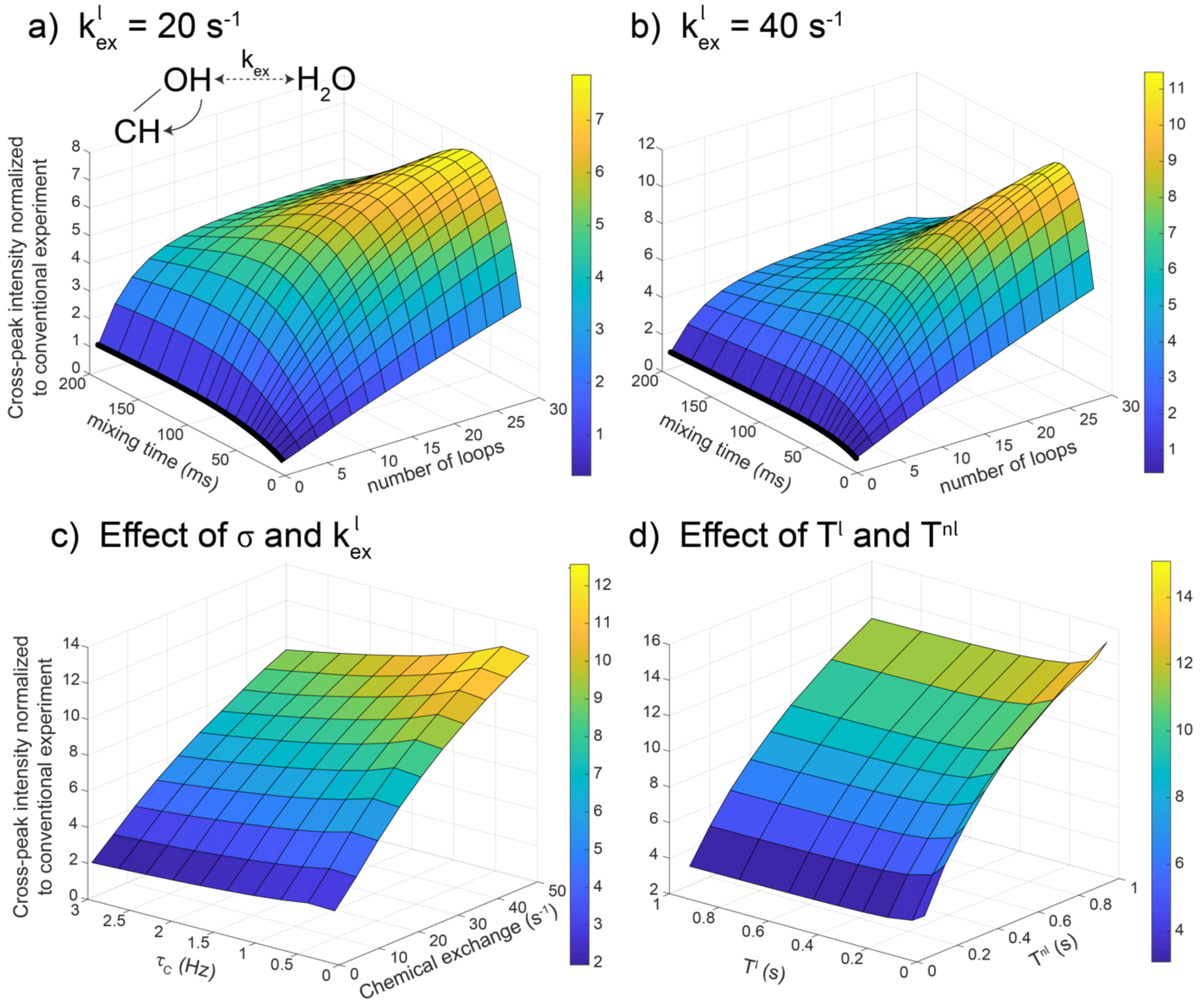

Supporting Figure S2. L-PROSY buildups according to Bloch-McConnell equations with respect to NOE mixing and number of loops for exchange rates of **a)** 20 s^{-1} and **b)** 40 s^{-1}. Bolded curves represent the buildups in conventional experiments; tumbling time τ_C was assumed to be 0.2 ns and internuclear distance r was 2.3 Å. c) Dependence of the maximum L-PROSY enhancement ε predicted with respect to the rotational tumbling time and the chemical exchange rate with the solvent. d) Effect of the labile and non-labile relaxation times in determining L-PROSY enhancements, assuming an exchange of 40 s^{-1}. In all cases the aqueous pool was assumed ~500-fold larger than the solute; unless explicitly varied, $T_1^l = 0.5\ s$, $T_1^{nl} = 0.8\ s$ and $T_1^{H_2O} = 2\ s$ exemplifying small molecules' relaxation rates. See text for more details.

3) L-PROSY brings substantial enhancements to 3D TOCSY-fHSQC correlations. The sequence shown in Figure 3a was used to obtain 3D H_O-H_C-^{13}C correlations for 25% labeled glucose sample utilizing NUS. Figure S3 illustrates resulting 3D cube with assignments provided in ^{13}C -^{1}H plane. Considering enhancements discussed in the main text achieved for

glucose under the same conditions, acquisition of corresponding conventional 3D diagram would take >10x longer, which is a substantial difference in required instrument time.

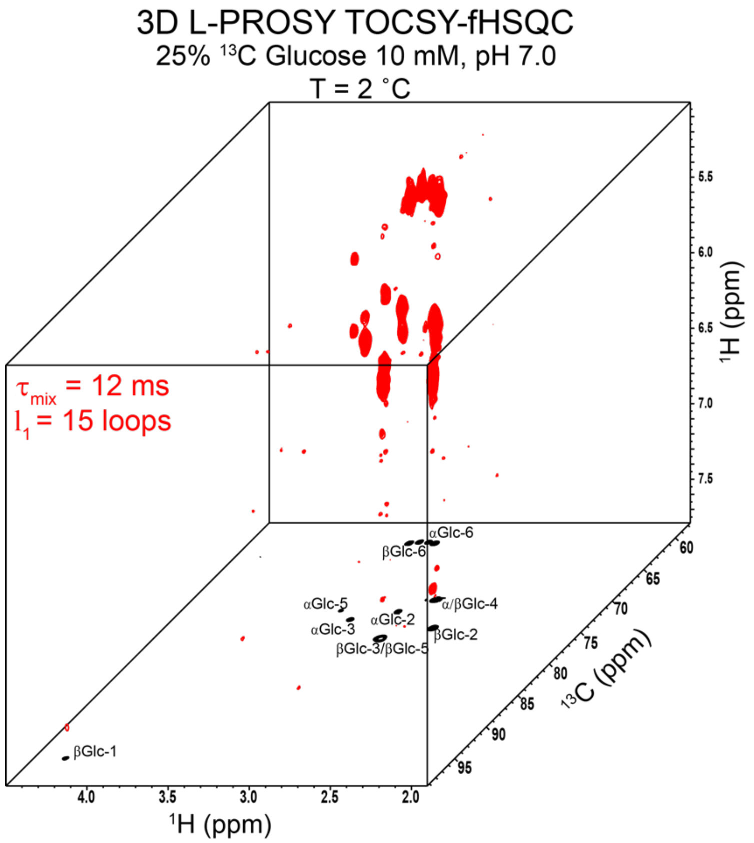

Supporting Figure S3. 3D cube representing the correlations between hydroxyl protons (F1), aliphatic protons (F3) and their neighboring carbon (F2) atoms, acquired using 3D L-PROSY TOCSY fHSQC. Experiment is acquired using 8% NUS sampling in 15 hours using rather long relaxation delay of 2s for aliphatic protons to recover. Considering typical >3-fold gain achieved in L-PROSY (corroborated with the same sample of Glucose using 2D L-PROSY), similar conventional 3D NOESY-HSQC experiment would require at least 5 days longer acquisition time to achieve similar sensitivity. Plane F_2F_3 is showing ^{13}C-^{1}H projection, with included assignments.

4) Magnetic field effects on L-PROSY glycan spectra. Supporting Figure S4 complements Figure 4, by experimentally examining the effects of the external field on selective NOESY and L-PROSY NOESY spectra collected on a Sia_4 sample. Notice that whereas a single-loop selective NOESY experiment fails to reveal any cross-peaks at 14 °C and 600 MHz, in L-PROSY these cross-peaks start to appear. The selective NOESY spectrum at 1 GHz is slightly better than at 600 MHz, but even further advantages arise when L-PROSY is incorporated. In practice, the optimal L-PROSY conditions will be an interplay between chemical exchange rates and ensuing broadening complicating the selective pulses in this experiment. This is evidenced from the middle and bottom panels of Figure S4: higher quality spectra are obtained at 5 °C than at 14 ˚C, despite the smaller L-PROSY enhancements observed (indicated for several peaks). The absolute signal-to-noise ratio (SNR) of the cross-peaks can be appreciated from the various projections extracted for the same resonances in these spectra.

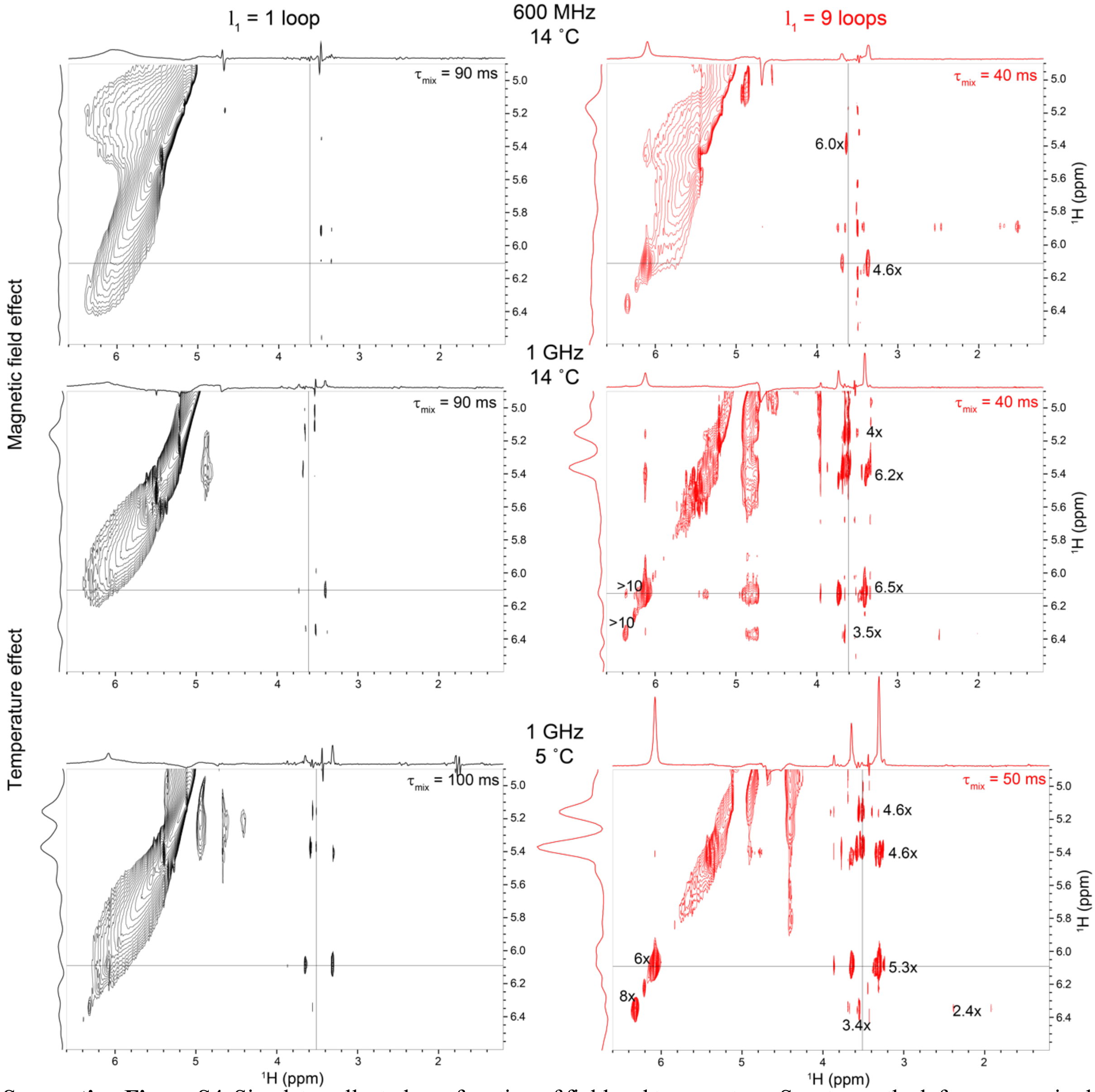

Supporting Figure S4. Sia4 data collected as a function of field and temperature. Spectra on the left were acquired with one loop, representing the results of a conventional, frequency selective NOESY experiment. Panels on the right show L-PROSY counterparts acquired with 9 loops. F1 and F2 projections are extracted from the same resonances in all spectra (accounting for temperature-induced shifts) and demonstrate clearly the trend in L-PROSY enhancements and absolute SNR of the experiment. Enhancements for several diagonal peaks and their corresponding cross-peaks are indicated in the spectra at 1 GHz, while at 600 MHz they could not be measured with certainty. All spectra were acquired with slightly different parameters, as these were optimized for each temperature and field.

5) TOCSY correlations of Sia4 glycan. A conventional 1 GHz TOCSY spectrum recorded on Sia4 at 5 °C (Figure S5a), displays correlations from the aliphatic protons (evolved in the indirect dimension, F_1) to the directly detected OHs (in F_2). Their diagonal counterparts are greatly attenuated due to exchange with the solvent over the course of the TOCSY mixing time; hence, this chemical shift range is not shown along the F_1 axis. In contrast, an L-PROSY TOCSY spectrum relying solely on the OH's t_1 evolution (Figure S5b), generates cross-peaks

to all ^{1}Hs in the structure. Notice that as a consequence of this, x and y axes (F_1 and F_2) in the spectra of Figures S5a and S5b were “flipped”; this leads to different spectral resolution along the F_1/F_2 domains between conventional and L-PROSY-based TOCSY, and to a strong water cross-peak in the conventional acquisition. Multiple short-range TOCSY aliphatic/hydroxyl cross-peaks are coincident in both experiments, even if they are enhanced by ~1.5-4x factors in the L-PROSY version. Moreover, looping the short isotropic mixing processes reveals long-range *J*-mediated correlations which are absent in conventional acquisitions, including cross-peaks for IOH7-I5 protons separated by five covalent bonds, and for IIIOH7-III8 and IIIOH7-III6 peaks among protons that are separated by four covalent bonds. Such long-range correlations involving the OHs, enable the complete spin-system assignment for this glycan.

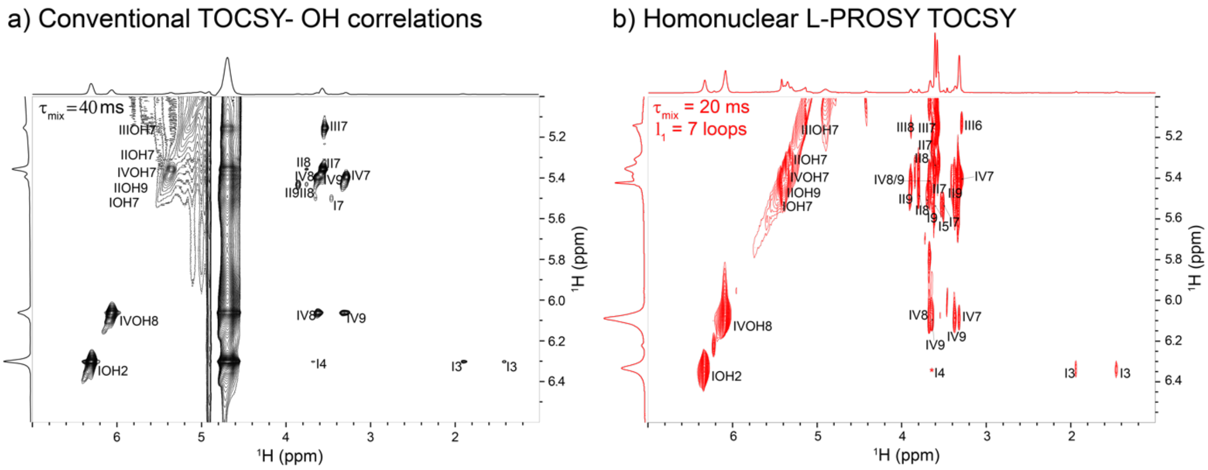

Supporting Figure S5. Conventional and L-PROSY TOCSY and NOESY experiments acquired on Sia_4 at 5 °C and 1 GHz. a) involved a single DIPSI2 40 ms long mixing, which is the upper boundary when considering the fast OH's chemical exchange with water, b) looped a 20 ms DIPSI2 block 7 times. F_1 and F_2 dimensions in (a) and (b) were flipped in order to match the OH correlations detected on the two experiments. Shown in the spectra are the unambiguous assignment arising from the two experiments; shown on the top and side are skyline projections

6) L-PROSY NMR on Sia_4’s exchangeable amide hydrogens. Alongside OHs, Sia_4 possesses a number of labile amide sites that were enhanced by L-PROSY. This is verified in Supporting Figure S6, with a number of intra- and inter-residue L-PROSY NOESY correlations involving these protons. Cross-peaks between these amide and aliphatic protons (top) as well as correlations that extend these to aliphatic ^{13}C sites by 2D ^{13}C-^{1}H projections from L-PROSY NOESY-fHSQC at 14 °C, evidence these enhancements. Intra-residue cross-peaks between the amide protons and closest neighbors in positions 4, 5, 6, 7 and 11 (2.3 – 3.2 Å) are enhanced ~2x in L-PROSY vis-à-vis the conventional NOESY. On the other hand, this suffices to visualize correlations with protons that are further apart, such as those at positions 3, 8 and 9 (4.7 – 5.3 Å). Slightly larger (~2.5x) enhancements are observed for an

inter-residue cross-peak between INH and II3 which at a distance of ~5 Å. None of these are visible on the conventional spectra (not shown).

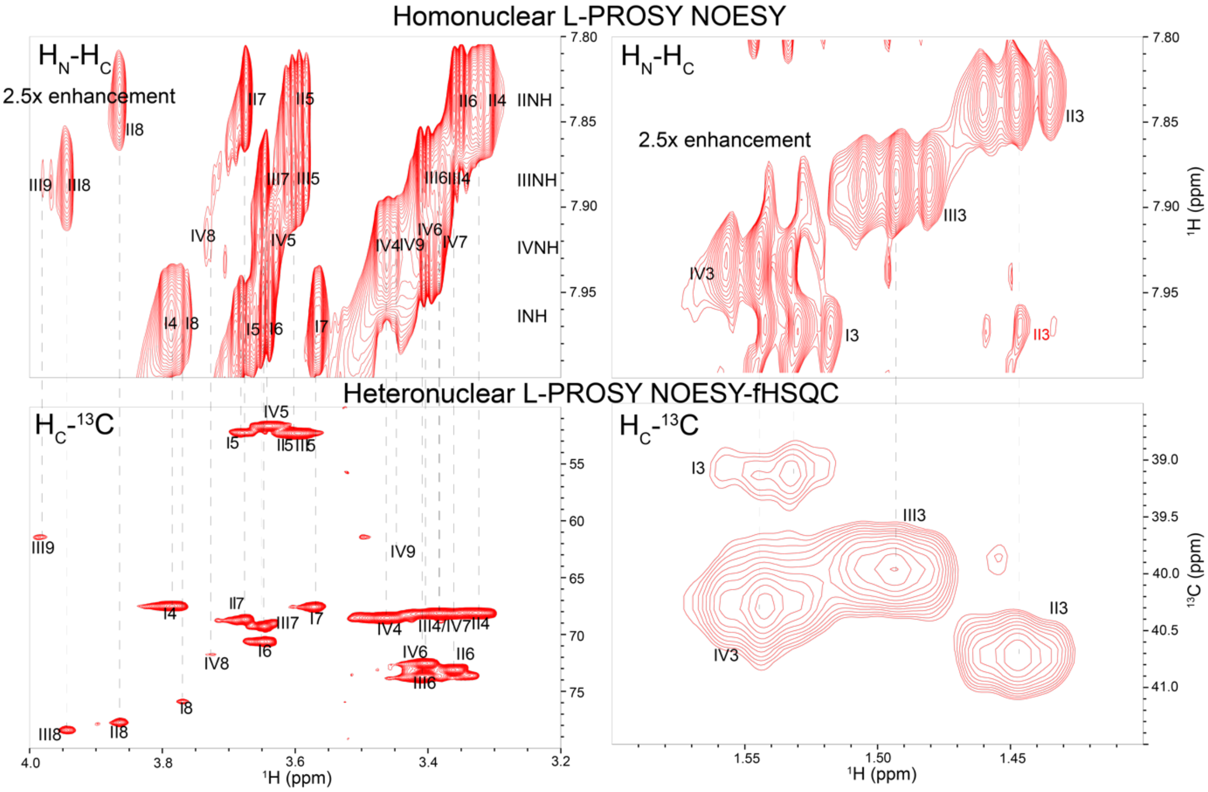

Supporting Figure S6. Top panels show amide-aliphatic proton cross-peak correlations arising in Sia_4 for two distinct chemical shift regions. Data were acquired with a homonuclear L-PROSY NOESY experiment by looping 7 times a 140 ms NOE mixing period. Bottom panels show corresponding ^{13}C correlations acquired with L-PROSY NOESY-fHSQC by evolving only $HzCx$ single-quantum coherences in a post-NOESY fHSQC block. Aliphatic protons at positions 8, 9 and 3 are furthest from the amide protons, hence showing the highest enhancements. Notice the internuclear correlation observed between an amide proton of the first residue and the aliphatic proton at position 3 of the second residue. Spectra were acquired at 14 °C on a 1 GHz Avance NEO.

7) Probing for aggregation in Sia_4. Because H-bond and NOE correlations may be misinterpreted if the molecule aggregates in solution, we performed a series of DOSY experiments as a function of concentration and temperature. Supporting Figure S7 summarizes the results of these measurements, which by showing no dependence on sample concentration indicate a lack of aggregation.

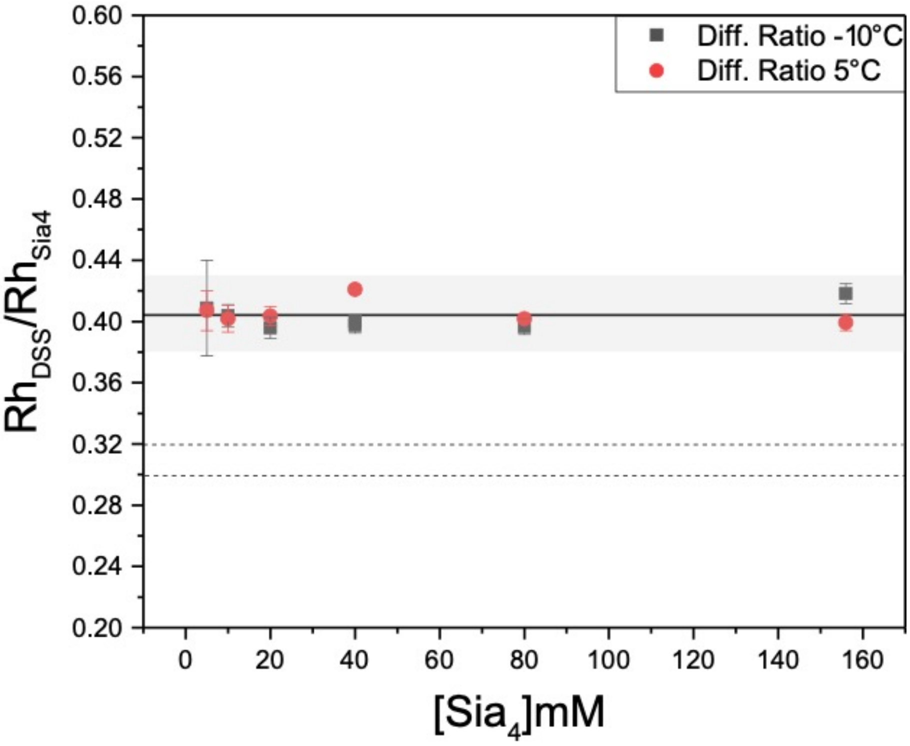

Supporting Figure S7. Translational diffusion of Sia_4 relative to DSS (reference molecule) as a function of concentration probed at -10 and 5 °C (red circles and black squares, respectively). The results show a consistent ratio throughout the working concentration range, with values at the highest concentration (ca. 160 mM) indistinguishable from the values obtained at the lowest concentration tested (5 mM). The grey box indicates the standard deviation from the mean diffusion ratio values measured at both temperatures and all Sia_4 concentrations. A persistent Sia_4 e.g. dimerization would result on s drop on Rh_{DSS}/ Rh_{Sia4} an increase in diffusion ratio as the concentration of Sia_4 increases, behavior that is not detected in any of the conditions used in this study. The expected drop in Rh_{DSS}/ Rh_{Sia4} was calculated to be between 20-30% in the measured ratio (shown by dashed lines in the figure). The expected decrease in Rh_{DSS}/Rh_{Sia4}, was calculated by simulating translational diffusion values for elongated and compact Sia_4 models in their monomeric and dimeric forms, as well as for DSS using HYDROPRO.[12]

8) HSQC-NOESY of Sia_4 at -10 °C. These ancillary data (Supporting Figure S8) were acquired with the hsqcetf3gpno Bruker sequence with minor modifications to improve water filtering. The carrier frequency, spectral window, number of points for the direct and indirect dimensions were set to: 4.7 ppm, 50 ppm; 10 ppm, 58 ppm; 4096, 2048, respectively. The data were acquired with 30% NUS and processed in NMRPipe employing SMILE for data reconstruction. The spectrum shown in Figure S8 was collected in ~64 h (256 scans per t_1 point). Intra-ring NOEs were utilized for distance calibration. The carrier frequency; spectral window; number of points for the direct and indirect dimensions were set to 4.7 ppm, 65 ppm; 10 ppm, 30 ppm; 8192, 256, respectively. 128 scans/t_1 point were used. HSQC-NOESY experiments were acquired with various mixing times (100, 150 200, 250, 300 and 400 ms). NOE build up curves

were generated from these data, from which the optimal mixing times was selected to generate distances for structure calculation.

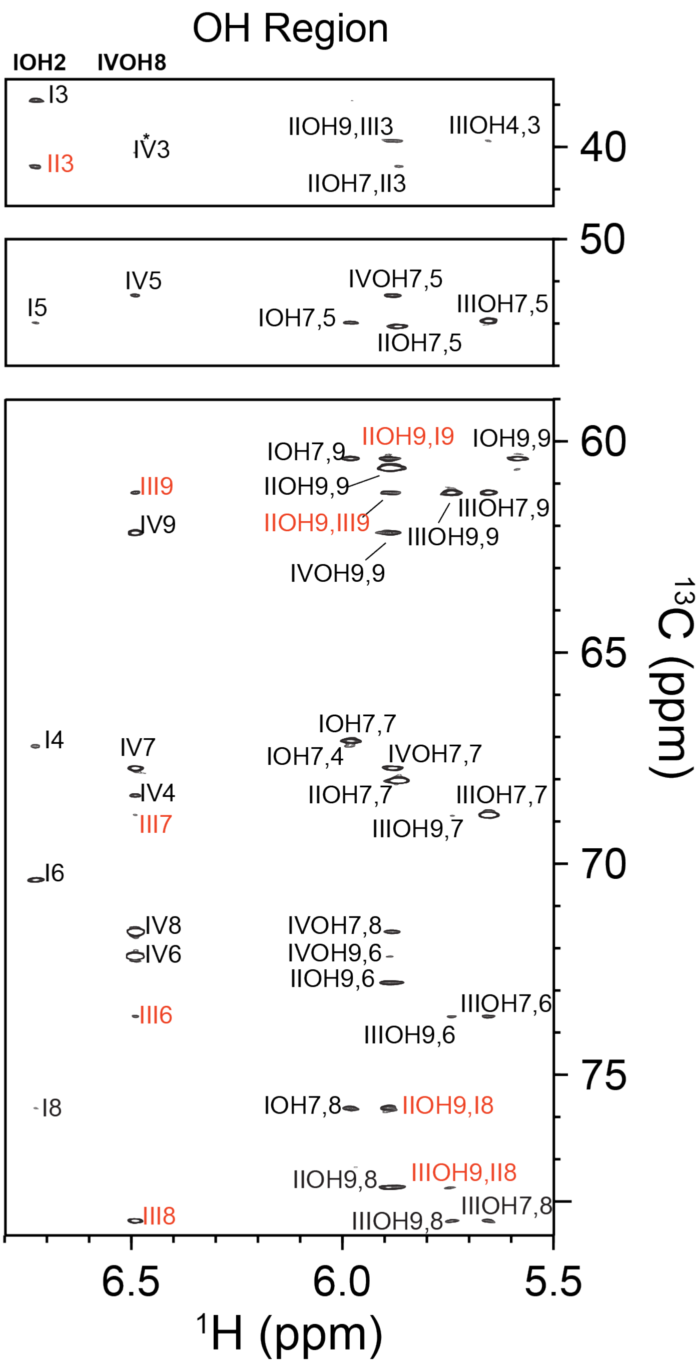

Supporting Figure S8. Fully assigned OH region of a 150 ms HSQC-NOESY collected at -10 °C on a 78.7 mM sample, in 90:10 H_2O:D_2O, pH 7.5 at 700 MHz. DSS was used as internal reference. Long-range correlations are labeled in red. The observed correlations are fully consistent with those detected by L-PROSY-NOESY experiments at 5 ˚C (see Table 1 and main text for further discussion).

9) *$^{3}J_{CH}$ measurements on Sia$_4$* Heteronuclear coupling constants were measured using the PIP-HSQMBC experiment. The PIP-HSQMBC was used as published[9] but with minor modifications for water signal removal through coherence selection. A final spectral resolution of 0.5 and 30 Hz/point were used for the ^{1}H and ^{13}C dimensions, respectively. Briefly, in-phase and anti-phase PIP-HSQCMBC experiments, optimized to observe 6 Hz $^{3}J_{CH}$, were collected

on a ca. 160 mM Sia4 sample containing 10% ^2H_2O and 0.1% DSS. The resulting experiments were added and subtracted to yield in-phase spectra with cross peaks with chemical shifts offset by the $^nJ_{CH}$. Coupling constants were determined by frequency difference between the offset cross peaks. Representative PIP-HSQCMBC spectra are shown in Supporting Figure S9.

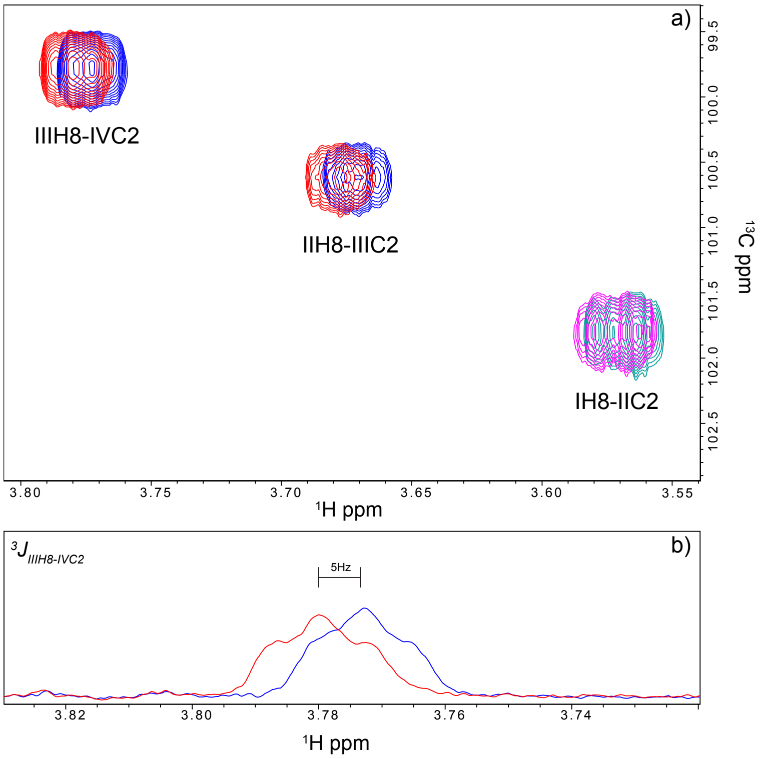

Supporting Figure S9. Set of PIP-HSQMBC spectra collected on a ca. 160 mM Sia4 sample on a 700 MHz instrument at -10 °C (a). The overlaid spectra with blue/teal and red/pink cross peaks were obtained by either subtracting or adding the anti-phase /in-phase PIP-HSQMBC spectra, respectively. Panel b shows resulting 1D projections from the IIIH8-IVC2 cross peaks shown in Panel a, with the corresponding measured $^3J_{HC}$.

Only the two set of six ^3J coupling constant values that were consistent with a defined torsion angle value were used for NMR structure calculation at -10 °C and at 5 °C. Based on the measured ^3J, the following torsion angle values were used: ω6 (H6-C6-C7-H7)= ± 90 ± 30° for residues I, II, III and IV and ω8(H7-C7-C8-H8)= 180 ± 30° for residues I and IV. We have also measured $^3J_{H8C2}$, which can be correlated to the ψ (H8-C8-O2'-C2'), yielding 2.9±0.5 Hz, 4.2±0.5 Hz and 5.0±0.5 Hz for residue I, II and III, respectively. These values remained fairly constant, withing the experimental error, for the temperature range tested (-10 °C, 5 °C and 37 °C), suggesting a similar conformational preference for these torsions over -10 °C to 37 °C temperature range. However, the ψ torsions were not restrained for structure calculation because the data cannot be unambiguously interpreted from a α2,8 Sia-specific Karplus-like parameterization (not available).

10) Simulated annealing results. Structures resulting from the SA calculations described in the main text, led to the results shown in Supporting Figure S10.

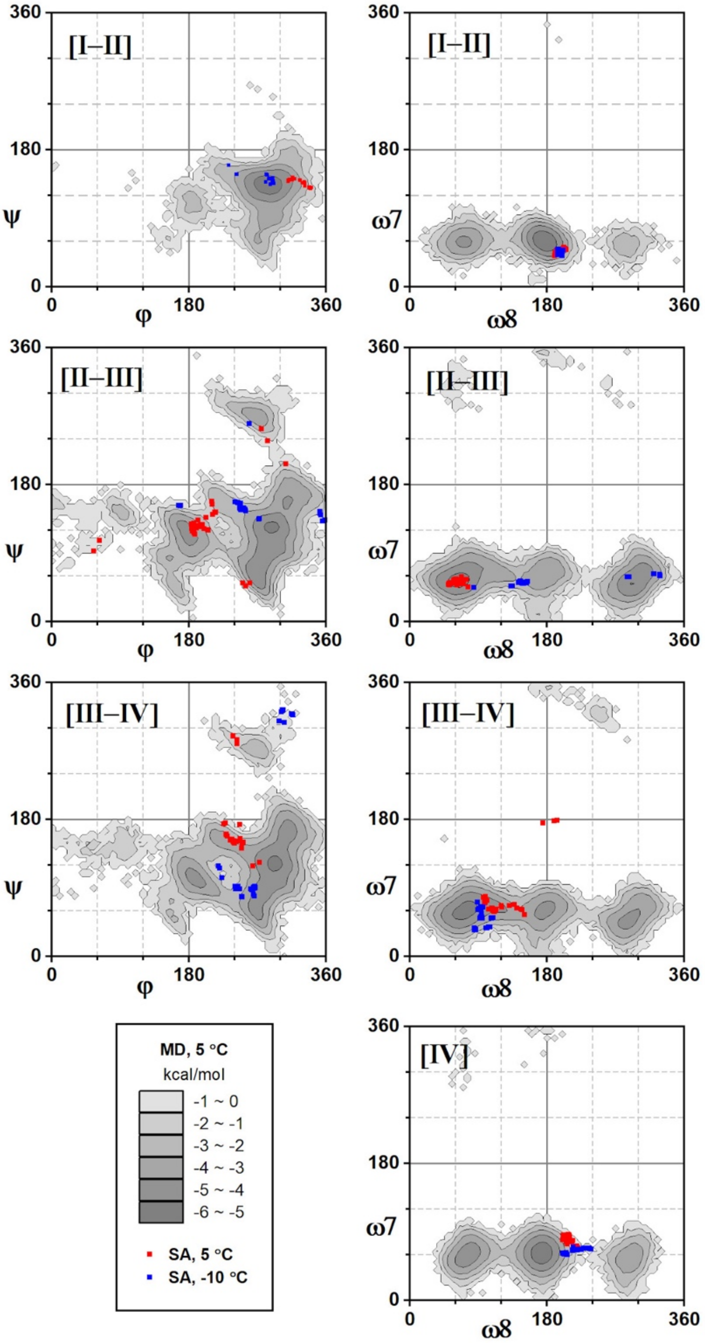

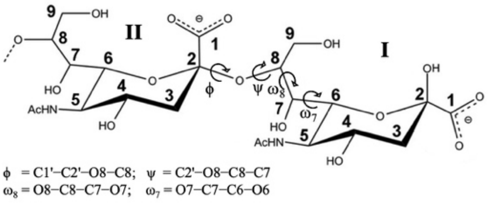

Supporting Figure S10. Values for the torsion angles adopted by the linkers between Sia residues for the structures shown in Figure 6a (Sia_4 NMR ensemble -10 °C, blue squares) and 6b (Sia_4 NMR ensemble 5 °C, red squares), overlayed over contour plots from the MD simulation at 5 °C. In some cases, such as ϕ for Residue I and ϕ and ψ for Residues II and III, the torsions change with temperature. The values for ω8 are temperature dependent mainly for Residue II, while in Residues I, III and IV ω8 is less affected. Note that most of the resulting torsion values while represented on the MD simulation do not coincide with MD energy minima, which might be due to experimental sampling limitations, simulated annealing bias or force-field imperfections. Most likely it is a combination of those factors, which our studies attempt to disentangle.

11) ***Sia₄ OH's chemical shift temperature dependence.*** The OH chemical shifts as a function of temperature were acquired with the hsqcdietgpsi Bruker sequence with minor modifications. A 10 ms of DIPSI2 isotropic mixing was used. The same acquisition parameters as for the HSQC-NOESY experiment were employed except that 512 complex points (32 scans/t_1) and uniform sampling were used instead. Data were acquired at -10, -5, 0, and 5 °C. Measured OH chemical shifts were plotted as a function of temperature and fitted to a linear function to yield the temperature coefficients with error representing fitting error.

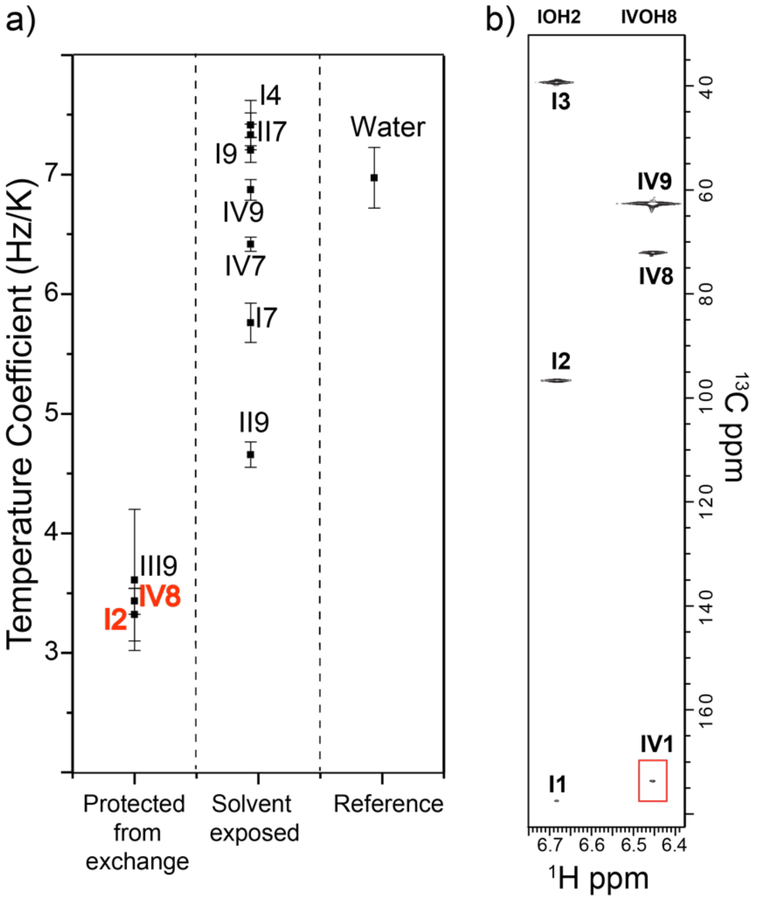

Supporting Figure S11. Experimental results supporting the presence of OH-based H-bonds in Sia_4. a) Measured temperature coefficients for Sia_4 OH groups. The OH groups found to have low temperature coefficients are (red labels) are those that stabilize motifs present Sia_4. The results observed for OH9 in residue III (labeled III9) are more inconclusive, as no evidence of III9's involvement in an H-bond is found. b) IOH2 and IVOH8 regions of a long-range HSQMBC experiment., showing a novel IVOH8-IVC1 through-H-bond correlation (red square) indicative of the IVOH8••O1 H-bond. No other long-range through-H-bond was detected in this experiment.

12) Hydrogen bond detection experiment. A long-range HSQMBC sequence[10] was used for H-bond detection, albeit with minor modifications to remove water interference. The carrier frequency, spectral window, number of points for the direct and indirect dimensions were set to: 4.7 ppm, 100 ppm; 10 ppm, 180 ppm; 4096, 1024, respectively. The spectra shown in Figure S11b was collected in ~42 h using 40% NUS (256 scans per t_1 point). A long-range delay of 62.5ms was used for $^{n}J_{CH}$ evolution. The used Poisson-gap schedule employed for data collection was generated with Schedule generator V3.0 (http://gwagner.med.harvard.edu/intranet/hmsIST/gensched_new.html).

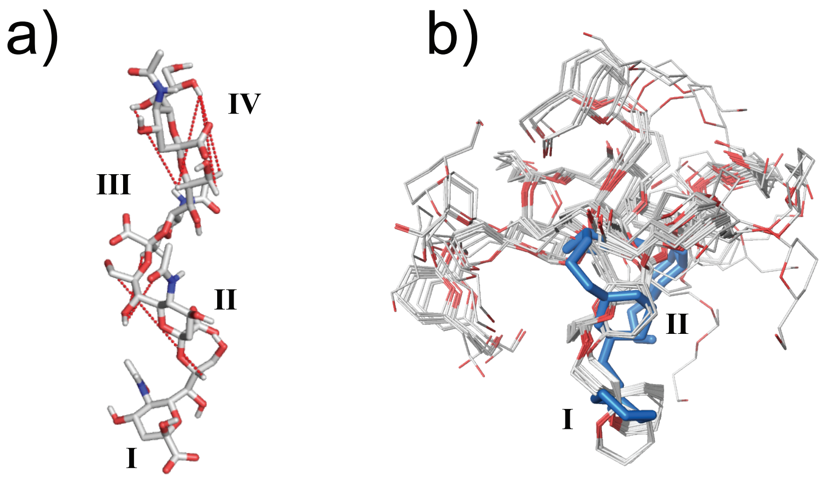

Supporting Figure S12. a) Previously proposed Sia_4 structure, involving an extended helical model inconsistent with the L-PROSY correlations shown with the dashed lines. **b)** NMR ensembles composed of 100 structures generated via simulated annealing, recalculated using data reported for the helical model[11] (Only the ring and linker chain heavy atoms are shown, with carbon and oxygen atoms shown in white and red, respectively; close to mean structures are shown in blue. Residue numbering is indicated by Roman numerals, where "I" indicates the "reducing" and "IV" the "non-reducing end", respectively.

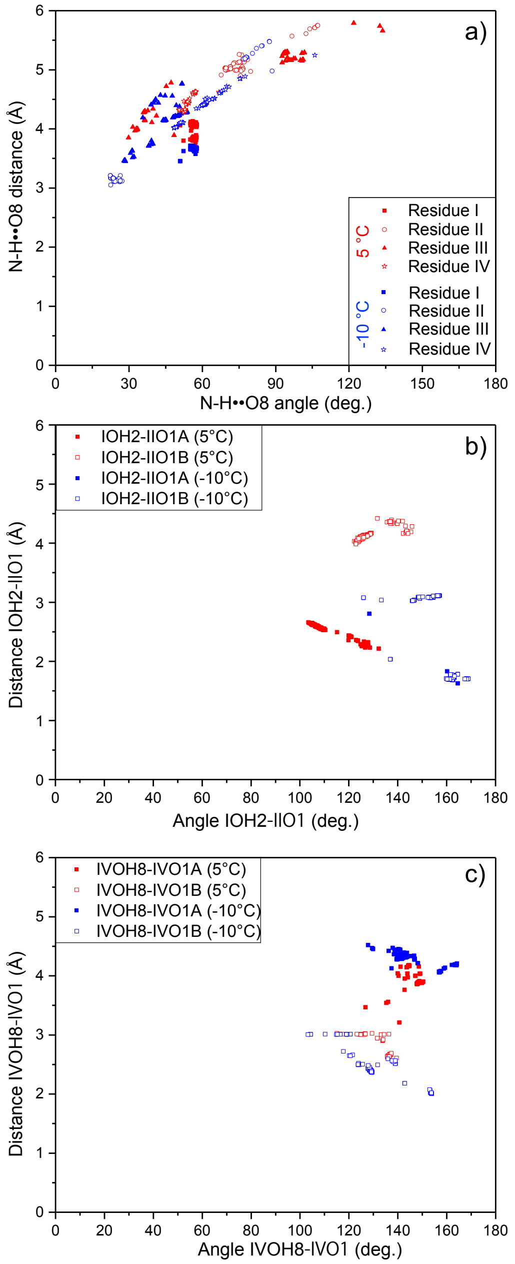

Supporting Figure S13. a) Plot of N5-H••O8 distance vs. angle for the NMR structural ensembles shown in Figures 6c and 6d (Blue and Red geometric figures, respectively) for each of Sia_4's residues I, II, III and IV, respectively. Irrespective of the temperature, the structural ensembles do not consistently fit a strong H-bond since the angles and distances between the donor and acceptor pairs are far from ideal –indicating that any interaction between donor and acceptor pairs would be week due to poor geometry. Conversely, based on consistent NMR and MD evidence, the NMR-derived structural ensembles from data collected at -10 °C and 5 °C, (depicted by blue and red geometric figures) are consistent with persistent IOH2-IIO1A/B (b) and IVOH8-IVO1A/B (c) H-bonds.

Table S1. NMR ensemble statistics from the models shown in Figure 6.

	**2012 data-derived ensemble**	**Sia_4 at -10 °C Revised ensemble (this study) with all restraints**	**Sia_4 at 5 °C (this study) with all restraints**
	(Figure S12b)	(Figure 6a)	(Figure 6b)
**Intra-residue long-range NOEs (>4 bonds away)**	None	30	17
**Sequential inter-residue NOEs**	29	38	31
**Non-sequential inter-residue NOEs**	None	6	0
**Torsion angle restraints**	6	6	6
**Hydrogen bond restraints**	None	2	2
**Averaged RMSD to closest to mean structure (I to II)**	0.6 ± 0.7 Å	0.41 ± 0.04 Å	0.5 ± 0.1 Å
**Averaged RMSD to closest to mean structure (I to IV)**	3 ± 1 Å	0.9 ± 0.5 Å	1.4 ± 1.0 Å

References

(1) Ernst, R. R.; Bodenhausen, G.; Wokaun, A.; *Principles of Nuclear Magnetic Resonance in One and Two Dimensions*; Clarendon Press, Oxford, **1989**.

(2) Abragam, A.; Goldman, M. Principles of Dynamic Nuclear Polarisation. *Rep. Prog. Phys.* **1978**, *41* (3), 395.

(3) Goldman, M. *Quantum Description of High-Resolution NMR in Liquids*; Clarendon Press, Oxford, 1990. https://doi.org/10.1063/1.2810567.

(4) Hogben, H. J.; Krzystyniak, M.; Charnock, G. T. P.; Hore, P. J.; Kuprov, I. Spinach - A Software Library for Simulation of Spin Dynamics in Large Spin Systems. *J. Magn. Reson.* **2011**, *208* (2), 179–194. https://doi.org/10.1016/j.jmr.2010.11.008.

(5) Kuprov, I. Diagonalization-Free Implementation of Spin Relaxation Theory for Large Spin Systems. *J. Magn. Reson.* **2011**, *209* (1), 31–38.

(6) Kuprov, I. Large-Scale NMR Simulations in Liquid State: A Tutorial. *Magn. Reson. Chem.* **2018**, *56* (6), 415–437. https://doi.org/10.1002/mrc.4660.

(7) Novakovic, M.; Cousin, S. F.; Jaroszewicz, M. J.; Rosenzweig, R.; Frydman, L. Looped-PROjected SpectroscopY (L-PROSY): A Simple Approach to Enhance Backbone/Sidechain Cross-Peaks in 1H NMR. *J. Magn. Reson.* **2018**, *294*, 169–180.

(8) Levitt, M. H. *Spin Dynamics: Basics of Nuclear Magnetic Resonance*; Wiley, 2nd edition **2008**.

(9) Castañar, L.; Saurí, J.; Williamson, R. T.; Virgili, A.; Parella, T. Pure In-Phase Heteronuclear Correlation NMR Experiments. *Angew. Chem.* **2014**, *126* (32), 8519–8522. https://doi.org/10.1002/ange.201404136.

(10) Williamson, R. T.; Buevich, A. V.; Martin, G. E.; Parella, T. LR-HSQMBC: A Sensitive NMR Technique to Probe Very Long-Range Heteronuclear Coupling Pathways. *J. Org. Chem.* **2014**, *79* (9), 3887–3894. https://doi.org/10.1021/jo500333u.

(11) Battistel, M. D.; Shangold, M.; Trinh, L.; Shiloach, J.; Freedberg, I. Evidence for Helical Structure in a Tetramer of α 2-8 Sialic Acid: Unveiling a Structural Antigen. *J. Am. Chem. Soc.* **2012**, *134*, 8–11.

(12) Ortega, A.; Amorós, D.; García de la Torre, J. Prediction of hydrodynamic and other solution properties of rigid proteins from atomic- and residue-level models *Biophys. J.* **2011**, *101*, 892-898.
